# Overview of myrmecological studies and a checklist of the ants (Hymenoptera, Formicidae) of the Democratic Republic of Congo

**DOI:** 10.3897/BDJ.12.e132915

**Published:** 2024-10-18

**Authors:** Hervé Ambakina, Kiko Gómez Abal, Benoit Guénard, Jean-Claude Monzenga, Evan P. Economo, Wouter Dekoninck

**Affiliations:** 1 Institut Facultaire des Sciences Agronomiques de Yangambi. Laboratoire d’Entomologie Appliquée et Fonctionnelle (LENAF), Yangambi, Congo Institut Facultaire des Sciences Agronomiques de Yangambi. Laboratoire d’Entomologie Appliquée et Fonctionnelle (LENAF) Yangambi Congo; 2 Independent Researcher, Garraf, Barcelona, Spain Independent Researcher Garraf, Barcelona Spain; 3 School of Biological Sciences, The University of Hong Kong, Pokfulam Road, Hong Kong, Hong Kong, China School of Biological Sciences, The University of Hong Kong, Pokfulam Road, Hong Kong Hong Kong China; 4 IFA-YBI, Kisangani, Democratic Republic of the Congo IFA-YBI Kisangani Democratic Republic of the Congo; 5 Okinawa Institute of Science and Technology, Kunigamigun, Okinawa, Japan Okinawa Institute of Science and Technology Kunigamigun, Okinawa Japan; 6 Royal Belgian Institute for Natural Sciences, Brussels, Belgium Royal Belgian Institute for Natural Sciences Brussels Belgium

**Keywords:** Formicidae, annotated list, distribution, Tropical Central Africa, Global Ant Biodiversity Informatics, species list

## Abstract

The production of species checklists is fundamental to setting baseline knowledge of biodiversity across the world and they are invaluable for global conservation efforts. The main objective of this study is to provide an up-to-date extensive checklist of the ants of the Democratic Republic of Congo (DRC), the largest country in sub-Saharan Africa, based on available literature to serve as a foundation for future research and ant faunistic developments. We gathered the literature available to us, most of it compiled from the Global Ant Biodiversity Informatics (GABI) Project and treated the data to province level when possible. We also offer insight into who, when and where contributions have emerged to the current knowledge of the ants of the DRC and each of its 26 provinces. The current list is restricted to valid species and subspecies, discarding morphospecies and some misidentified taxa. The list comprises eight subfamilies, 64 genera and 736 species, the highest species diversity for a country located within the Afrotropical realm.

## Introduction

The Democratic Republic of Congo (DRC) is the largest country in sub-Saharan Africa, ranking 11th in the world. Its size is comparable to western Europe with a surface of 2.3 million km^2^. It is divided into 26 provinces, ranging in size from Kasaï Oriental (9,481 km^2^) to Tshopo (199,567 km^2^). More than half of the country is covered by rainforest, representing the second largest forest massif globally after the Amazon (Nshimba 2008) with more than 1.08 million km^2^ (Devers 2007) and hosting a high level of botanical diversity with a total of 970 species of trees identified (Nshimba 2008, Ambakina 2022). DRC forests range from dense forests (semiper, semi-evergreen, swamp and secondary) to open forests (Zambezian and Sudanian), representing 47% of African forests (Achille et al. 2021, Alongo et al. 2022). Its ecological diversity also includes central river basins, wooded savannah, grassy savannah and mountain vegetation in the east, from Mayumbe to the south-west (Van Wambeke et al. 1956).

**Figure 1.**
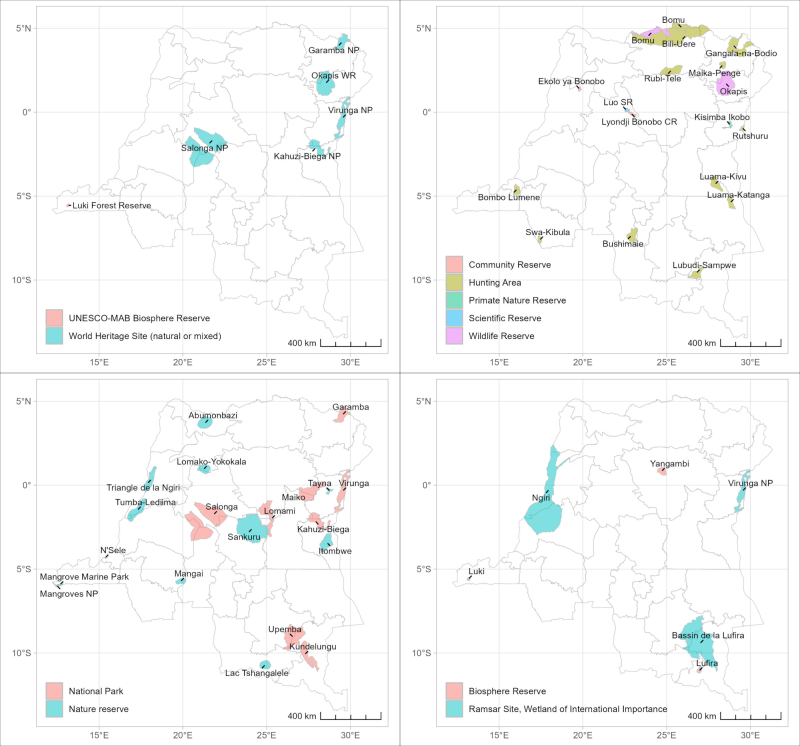
Protected areas in DRC. Prepared by the authors, based on data from UNEP-WCMC and IUCN (UNEP-WCMC and IUCN 2024).

Some of this exceptional diversity is protected under different entities (Suppl. material 1, Fig. 1) with 13.9% of the total surface of DRC protected in 2022 (World Bank Data).

These forests are, however, under threat. From 2001 to 2022, DRC lost 0.184 M km^2^ of tree cover, of which one third (0.06 M km^2^) was primary rainforest (Global Forest Watch 2024), equivalent to a 9.2% decrease. This represents 4% of the world’s total loss.

Afrotropical ant taxonomy is in a very weak state and the majority of the most diverse genera have never been revised (Robertson 2000, Gómez et al. 2023). This deeply affects our understanding of ant diversity, as most species are not being properly identified and are listed as morphospecies in most publications. The estimated number of ant species present in the Afrotropical Region is expected to double its current numbers (Robertson 2000). The DRC is expected to have a highly diverse ant fauna. Global assessments on the unknown diversity for the region cite DRC as one of the 25 world regions with the greatest hidden diversity measured in number of not yet found genera (Guénard et al. 2012). The mountainous North-East of the country has also been predicted as unexplored ant rarity centres that shall be revealed with future sampling (Kass et al. 2022).

With this in mind, the main objective of this work is to establish the current knowledge of the ants of the DRC at a provincial scale to help further ant research and stimulate researchers to mitigate the existing gap in ant diversity and taxonomy in the region.

## Material and methods

The present list is based on published records only. The main source of information is the extensive Global Ant Biodiversity Informatics (GABI) database (Guénard et al. 2017). As of February 2024, the Afrotropical database compiles 39,232 records of which 4,858 are from DRC. We filtered the records for identified valid species and discarded any records of morphospecies due to the lack of nomenclatural uniformity amongst the different collections. Hence, again, the numbers presented here should be taken with caution, especially for the genera that have never been revised, while, due to the limited sampling efforts conducted around the country, the true ant diversity is probably underestimated. Records have been individually refined to province level whenever the available information has made it possible. Distribution for each species is offered by province, except for the species that were cited only to country level.

Endemicity was assessed with the full Afrotropical database, covering species for each country at province level. Thus, we consider a taxon as “endemic” to the DRC when we could not find any other citation for that particular taxon in any other country. Assessing endemism in the Afrotropics, though, is a fraught exercise due to the generalised lack of sampling and poor taxonomy state in the region (Gómez et al. 2023) and should be interpreted with caution.

Codes used for provinces are the ISO 3166-2 without the country designator (e.g. TO instead of CD-TO). These are: BC: Kongo Central, BU: Bas-Uelé, EQ: Equateur, HK: Haut-Katanga, HL: Haut-Lomami, HU: Haut-Uelé, IT: Ituri, KC: Kasai Central, KE: Kasai Oriental, KG: Kwango, KL: Kwilu, KN: Kinshasa, KS: Kasai, LO: Lomami, LU: Lualaba, MA: Maniema, MN: Mai-Ndombe, MO: Mongala, NK: Nord-Kivu, NU: Nord-Ubangi, SA: Sankuru, SK: Sud-Kivu, SU: Sud-Ubangi, TA: Tanganyika, TO: Tshopo, TU: Tshuapa.

Entomological collections cited: CASC: California Academy of Sciences, California, USA. KGAC: Kiko Gómez Abal Collection, Spain. MRAC: Royal Museum for Central Africa, Tervuren, Belgium. RBINS: Royal Belgian Institute of Natural Sciences, Brussels, Belgium. UCDC: R.M. Bohart Museum of Entomology, University of California Davis, California, USA.

Data analysis was performed in R (v. 4.2.3, R CORE TEAM (2023)) to filter, homogenise and summarise the information. Packages used were *ggbreak* (Xu et al. 2021), *ggplot2* (Wickham 2016), *ggspatial* (Dunnington 2023) and *sf* (Pebesma and Bivand 2023) for charting and mapping; and *googlesheets4* (Bryan 2023), *stringr* (Wickhan 2022), *stringi* (Gagolewski 2022), *tidyverse* (Wickham et al. 2019) and *writexl* (Ooms 2023) for data analysis. Nomenclature is up to date following Bolton (Bolton 2023).

## Data resources

Sampling effort was assessed with the digitised collections available online (AntWeb, Antmaps), two Belgian Federal Institute collections (MRAC, RBINS) and one private collection (KGAC). The total number of records including identified and not identified samples exceeds 98,000 for the Afrotropical Region, 3,088 collected in DRC. We must emphasise that these data were used only to analyse sampling effort and not the species list, which is based on published records.

## Results

The number of recorded ant species and subspecies by genus and subfamily for the DRC, as well as their status within the Afrotropical realm is presented in Table 1. In total, eight subfamilies, 64 genera and 736 species, include 156 endemic were reported in the literature from the DRC.

DRC is the African country with the highest number of recorded ant species (n = 736, Table 1). Species richness percentages by subfamily are similar to those of the Afrotropical Region (AntWeb, visited March 2024), with about half (48.9% vs. 52% for the realm) of the species in the Myrmicinae, followed by Formicinae (23.6% vs. 22%), Dorylinae (12.1% vs. 8%) and Ponerinae (10.9% vs. 11%), with these four subfamilies totalling around 95% of the species diversity recorded so far. Dolichoderinae (2.7%, 20 species) is the next in the list, with the other three subfamilies, Pseudomyrmecinae, Proceratiinae and Apomyrminae, being rather species-poor with nine, four and one species, respectively.

**Figure 2.**
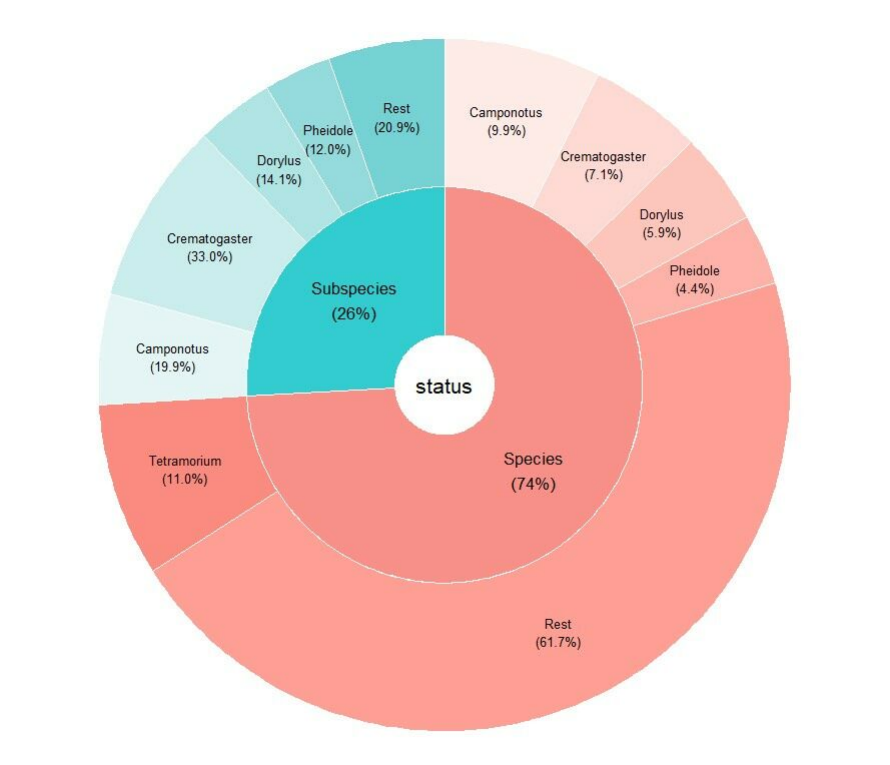
Ant species and subspecies proportion in DRC fauna.

**Figure 3.**
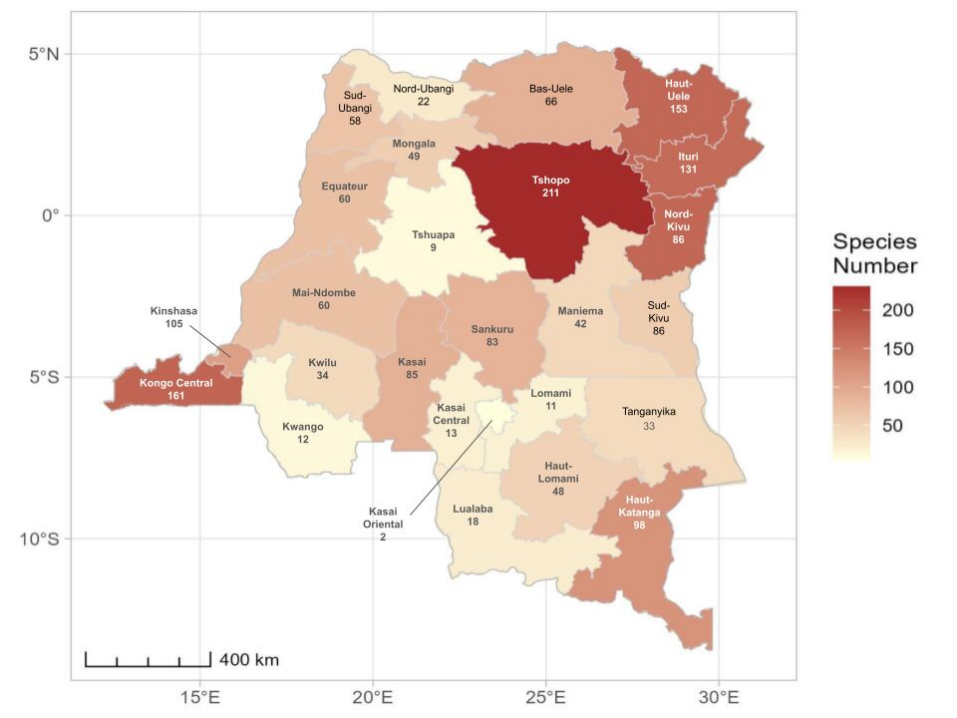
Ant species richness by province in DRC.

One in four of the records from DRC is a subspecies (Fig. 2 and Fig. 3), implying that their status has not been revised in modern times. Almost half of DRC diversity belongs to the five most diverse genera (Fig. 2). Of these five, only one (*Tetramorium*) has been revised (Bolton 1976, Bolton 1980, Bolton 1986, Hita Garcia et al. 2010, Hita Garcia and Fisher 2011, Hita Garcia and Fisher 2012, Hita Garcia and Fisher 2013, Hita Garcia and Fisher 2014, Mbanyana 2013, Mbanyana et al. 2018). However, despite these revisions, most of the genus still requires a lot of study, with likely 300+ additional undescribed species (Hita Garcia, pers. com.) Three genera (*Camponotus*, *Dorylus*, *Pheidole*) have more or less as many species as subspecies, while, in *Crematogaster*, two out of three named forms are subspecies. They include four-fifths of the subspecies known for the country. In these four genera, the more recently described species (*Pheidolechristinae*, *P.glabrella* and *P.setosa*) belong to the recently-revised *Pheidolepulchella* group (Fischer et al. 2012). Additionally, *Dorylusniarembensis* (van Boven 1972) is described from a single queen. All the other species were described before 1945.

Current diversity by province (Fig. 3) shows that most of the territory remains unexplored, with some provinces covered in dense rainforest, such as Tshuapa with only nine species cited.

**Figure 4.**
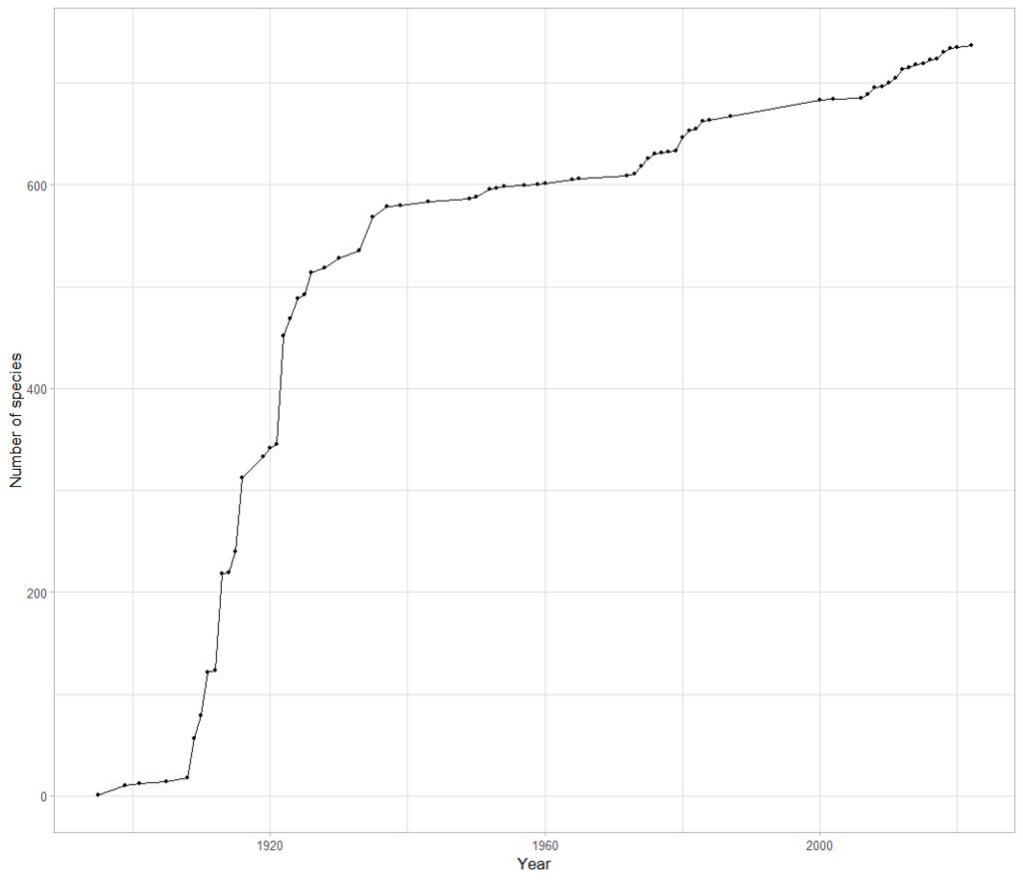
Accumulated ant species recorded in literature in DRC per year (1895-2024).

Knowledge timeline of DRC ants also reflects the lack of recent scientific activity (Fig. 4). Wheeler’s works (Wheeler 1922a, Wheeler 1922b) can be considered as one of the major achievements in the history of myrmecology, particularly in DRC, adding 107 new occurrences to the country. These two publications, now published over a century ago, provide records for 624 occurrences in the country or more than one quarter of all the published occurrences for the DRC (2,305). Its significance in the Afrotropical myrmecology is hard to comprehend, as it cites a total of 2,863 occurrences for the current amount of 22,872, so more than 10% of the existing records of ants in the Afrotropical Region available in the literature. It also summarises all the available taxonomical knowledge and distribution data up to 1922, describing 59 new species and subspecies, including 39 in DRC.

**Figure 5.**
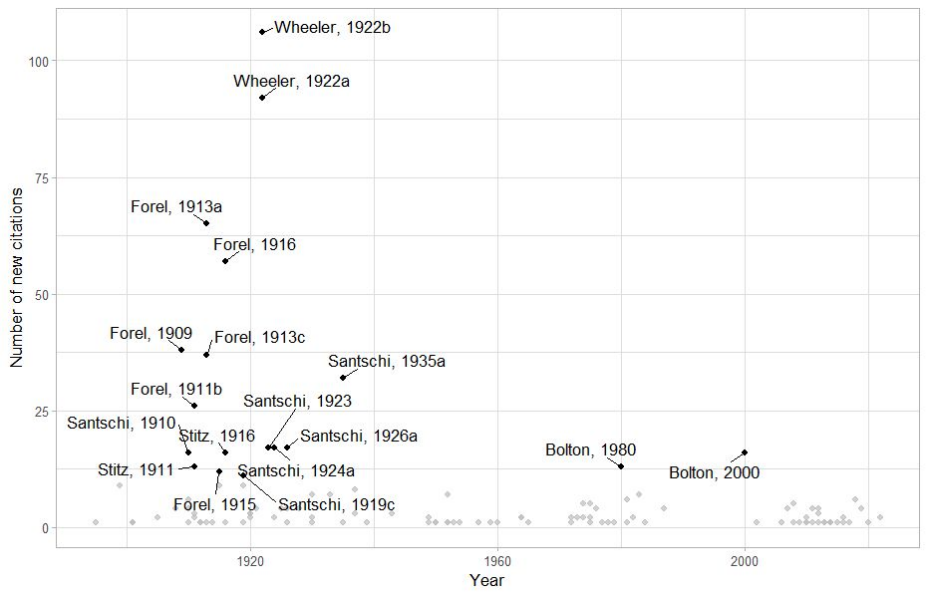
Number of ant species newly recorded in DRC, listed by author and year. References contributing to the addition of 10 or more species are highlighted.

Most of the ant diversity was already cited by 1950, with circa 600 species and almost all of it due to the efforts of early taxonomists: Forel, Santschi and Wheeler (Fig. 5).

In modern times (1970 to present), we ascertain that only 98 publications offer some data on the ant fauna of DRC, almost all of them in global taxonomic reviews, based almost completely on the collections stored in MRAC and RBINS. Only two publications are based on recent DRC sampling: Yamagiwa et al. (1991) deals with the stomach content of gorillas and Van De Perre et al. (2018) offers the results of collecting ants by hand in the Yangambi Forest in the context of a more general biodiversity assessment, citing 41 species and 23 morphospecies. It is also worth noting that the latest main publication (Bolton 2000) adding more than 10 newly-recorded species in DRC, dates back to nearly a quarter of a century.

**Figure 6.**
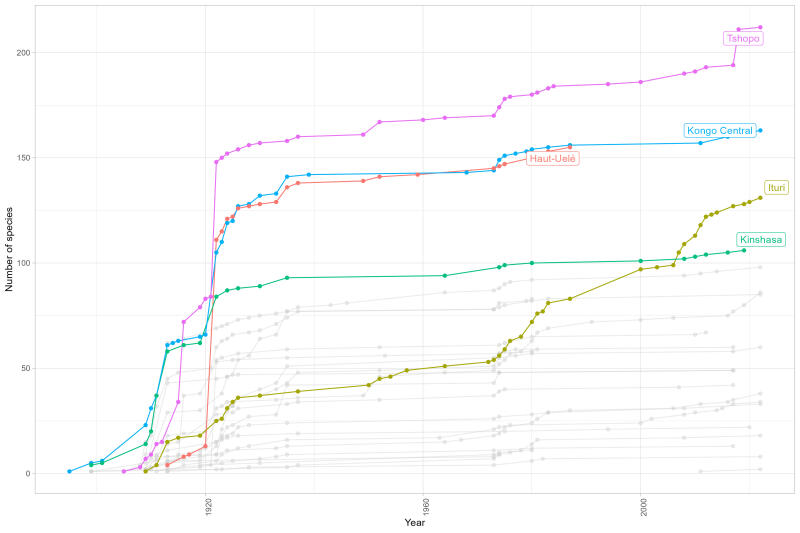
Ant species accumulation of newly-recorded species by year and province. The five most diverse provinces are highlighted.

**Figure 7.**
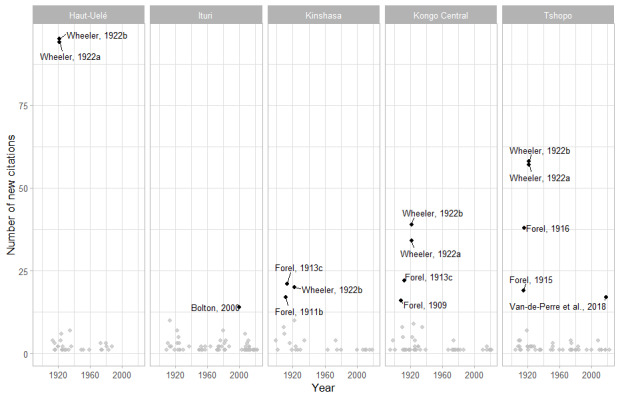
Number of new ant citations by reference and year for the five most diverse provinces.

The same analysis by province offers interesting insights (Figs 6, 7). The two provinces that form the “entrance gate” to the country by the Atlantic Ocean (Kinshasa and Congo Central) were the first sampled and early authors such as Santschi, Forel and Emery described most of their known diversity. The Provinces of Haut-Uelé and Tshopo were the main hunting grounds in the Wheeler expedition.

**Figure 8.**
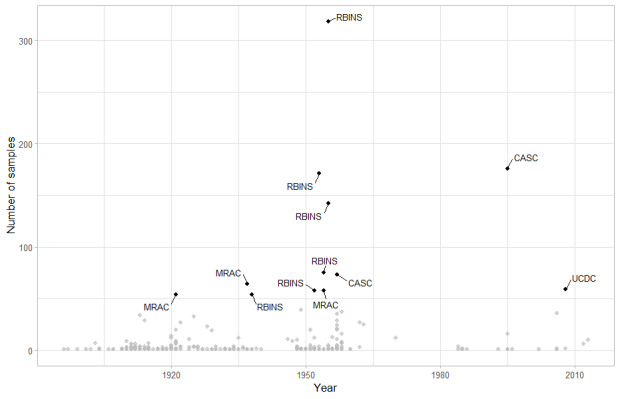
Available DRC ant material in digitised collections. Sources and years for collections including more than 50 specimens are highlighted.

A special case is the Ituri Province, where most of the diversity has been added by Barry Bolton since the 1980s. To understand this fact, we must explore the museum's material available. The main source for the Bolton (and subsequent authors) published taxonomic revisions comes from the field trips made more than one hundred years ago up to the 1930s (Bequaert, Burgeon, Collart, Mayné, Schouteden) or in the 1950s in the North-East of the country mostly by Belgian biologists, mainly in Ituri, Nord-Kivu and Sud-Kivu (Célis, Kekenbosch, Leach, Leleup, Ross, Saeger, Synave, van Boven, Vanschuytbroeck). Most of these collections are stored in the Royal Belgian Institute of Natural Sciences, Brussels (RBINS) and the Royal Museum for Central Africa, Tervuren (MRAC), both in Belgium. Less than 10% of these collections have been databased and, upon examination, comprise an estimate of at least 50,000 pins which are now being identified by the authors. The only exceptions to this rule are the specimens present in the CASC collected by Torti in 1995 in Epulu (Ituri), with 178 specimens and those present at UCDC from Wamba (Kwilu) collected by Heydon in 2006 and by Chapman in 2008 with a total of 51 specimens (Fig. 8).

**Figure 9.**
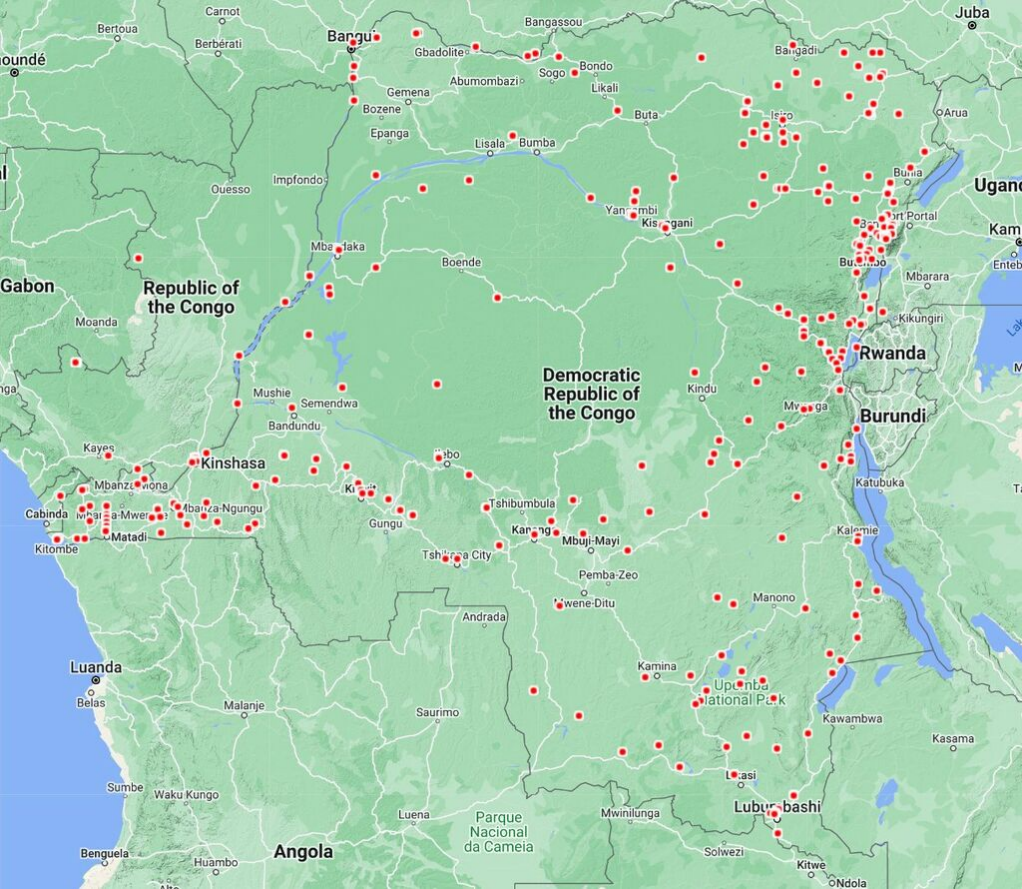
DRC collecting locations stored in AntWeb.org. (Accessed: March 2024).

Collecting sites can be approximated by the data in AntWeb.org, the largest repository of ant collection data on the internet. It holds data for 2,454 DRC specimens, of which 2,166 (88%) come from the collections at RBINS and MRAC from the 1920s to 1950s. More strikingly, Fig. 9 shows the large void existing in the DRC rainforest, with virtually no collections over an area of 450,000 km^2^, a size comparable to Spain or France in Europe, Cameroon or Ghana and Ivory Coast combined in Africa and one of the most diverse forests in the world.

## Species List

We have listed as NP (not present) four species that have been cited in the country, but represent obvious misidentifications dating back from more than 100 years ago: *Odontomachushaematodus* (Linnaeus, 1758), native to South America; *Oecophyllasmaragdina* (Fabricius, 1775) from Asia, probably misidentified for *Oecophyllalonginoda* (Latreille, 1802); *Tetramoriumdecem* Forel, 1913 has been re-identified as *Tetramoriumvenator*, Hita Garcia 2014 (Hita Garcia & Fisher 2014), except for the citation from Aba-Juba in Haut Uele Province (Weber 1952b), which we discard until the material can be seen; and *Tetramoriumkelleri* Forel, 1887 native to Madagascar (Table 2).

Two species are listed under DB (dubious). *Tetramoriumbicarinatum* (Nylander, 1846), cited for the country (Wetterer 2009), is cited as DB as no record for the Afrotropical Region has been properly identified, except for some in southern Africa (Francisco Hita García, pers. comm.). *Bothroponeracrassa* (Emery, 1877) belongs to a recently revised group and its presence could not be confirmed.

The current list, as we understand it and based on published data, comprises eight subfamilies, 64 genera and 736 species (Table 3, Suppl. material 1)

## Discussion

The current state of knowledge of ant diversity and taxonomy in the DRC is dismaying. Our results show that not only the limited taxonomic work conducted in the region may impair our understanding of ant diversity, as illustrated through the revision of parts of existing collections collected in the first half of 20^th^ century, but, in addition, it shows that the low sampling efforts in modern times (past 50 years) prevent a clear understanding of the diversity encountered within the country. This is highly problematic as new sampling methods have shown for the canopy forest (Basset 2001) or subterranean ants (Wong and Guénard 2017). As an example, the neotropical canopy forest harbours one third of the total ant diversity (Longino et al. 2002, Longino and Colwell 2020).

The economic and political circumstances in the country, along with the absence of local expertise explains most of the deficit of recent projects. As an example, the mountainous regions of Kivu and Ituri are thought to be extremely diverse (Kass et al. 2022), but also represent one of the most dangerous parts of the world.

Despite these facts, the country holds the highest number of recorded species in any country in the Afrotropical Region and our own investigations in the historic available material shows that several dozen species are to be added to the list. Additionally, records by province show that the country is mostly unexplored. The estimated number of species by province in tropical Africa should be between 150 and 300 for a reasonably sampled area and even more in primary rainforest (Weber 1943, Bernard 1953, Hita Garcia et al. 2009, Fotso-Kuate et al. 2015, Gómez, unpublished data). Only five out of 26 DRC provinces reach 100 cited species, but in at least two of these (Haut-Huelé and Ituri), our preliminary data in the Brussels’ collections reveal a much higher ant diversity. Other Provinces, such as Kwilu, Lomami and Tshuapa, do not reach 20 species.

Moreover, most of the current information has been added more than seventy years ago, with 601 species (80% of known diversity) already cited by the year 1952. Recent global taxonomic revisions have added citations new to DRC mainly based on curated material collected in the first half of the 20^th^ century, such as *Discothyreawakanda* (Hita Garcia et al. 2019), collected in 1963 in the Virunga National Park in North Kivu.

To the best of our knowledge, no modern intensive sampling collections in DRC have been conducted in recent decades. This is a tragic situation taking into account that the DRC area is similar to that of western Europe. The recent paper on geographic distribution of ants in western Europe (Wang et al. 2022) listed 747 species for the region, slightly higher than the DRC, but extensively sampled throughout the last century.

Exotic species may be underestimated in this list due to a lack of sampling. Only four species have been cited, but some usual suspects, such as *Tapinomamelanocephalum* (Fabricius, 1793), are likely to be present in Congolese cities.

While recent history is discouraging, we can only be optimistic about the long-term future of myrmecology in DRC, as only a glimpse of the real diversity is already astonishing. However, to reach this bright future, some objectives must be fulfilled. The first is to develop local expertise. This should comprise human resources in the universities and national institutes like Centre de Surveillance de la Biodiversité (CSB, DR Congo) and Institut Congolais pour la Conservation de la Nature (ICCN, DR Congo) to create and transmit this knowledge to the future generations. The second is building up material resources, mainly local reference collections. The challenge is making funds available to carry out sampling (both extensive and intensive) with modern techniques in the different ecosystems present in the country and to curate and preserve these collections locally and grant financial stability to local MSc and PhD students.

## Supplementary Material

8754BE11-CDB5-5C48-9A4F-503A808E6A34 10.3897/BDJ.12.e132915.suppl1 Supplementary material 1 List of protected areas in DRC Data type Table Brief description List of protected areas in DRC. Number in parenthesis represents the approximate area in km^2^. “*” denotes UNESCO wetland of international importance. File: oo_1088563.docx https://binary.pensoft.net/file/1088563 Ambakina H, Gomez K, Guénard B, Monzenga J-C, Economo EP and Dekoninck W

## Figures and Tables

**Table 1.** DRC number of ant species and subspecies by subfamily and genus, with their number of species considered as endemic or exotic.

**Subfamily**	**Genus**	**Species**	**Subspecies**	**Total**	**Endemic**	**Exotic**
APOMYRMINAE						
	*Apomyrma*	1		1		
**Total**		1		1		
DOLICHODERINAE						
	*Axinidris*	2		2		
	*Tapinoma*	2	1	3		
	*Technomyrmex*	15		15	1	
**Total**		19	1	20	1	
DORYLINAE						
	*Aenictogiton*	7		7	6	
	*Aenictus*	6	3	9	4	
	*Dorylus*	32	27	59	17	
	*Lioponera*	2		2		
	*Parasyscia*	3		3		
	*Simopone*	7		7	1	
	*Vicinopone*	1		1		
	*Zasphinctus*	1		1		
**Total**		59	30	89	28	
FORMICINAE						
	*Anoplolepis*	5		5		
	*Aphomomyrmex*	1		1		
	*Camponotus*	54	38	92	25	
	*Lepisiota*	19	9	28	12	
	*Nylanderia*	4		4	1	
	*Oecophylla*	1	5	6	1	
	*Paraparatrechina*	3		3		
	*Paratrechina*	1		1		1
	*Plagiolepis*	3		3	1	
	*Polyrhachis*	30		30	2	
	*Santschiella*	1		1		
**Total**		122	52	174	42	1
MYRMICINAE						
	*Atopomyrmex*	2		2		
	*Bondroitia*	1		1		
	*Calyptomyrmex*	7		7		
	*Cardiocondyla*	2		2		
	*Carebara*	12	4	16	10	
	*Cataulacus*	24		24	3	
	*Crematogaster*	39	63	102	38	
	*Cyphoidris*	1		1		
	*Dicroaspis*	2		2		
	*Melissotarsus*	2		2		
	*Meranoplus*	4		4		
	*Microdaceton*	1		1		
	*Monomorium*	20		20	2	1
	*Myrmicaria*	9	11	20	5	
	*Nesomyrmex*	3		3		
	*Pheidole*	24	23	47	7	
	*Pristomyrmex*	3		3		
	*Solenopsis*	2	3	5	2	1
	*Strumigenys*	33		33	3	
	*Syllophopsis*	1		1		
	*Tetramorium*	59		59	7	
	*Trichomyrmex*	4		4	1	1
**Total**		255	104	359	78	3
PONERINAE						
	*Anochetus*	8		8		
	*Bothroponera*	6		6		
	*Brachyponera*	1		1		
	*Centromyrmex*	7		7	1	
	*Euponera*	2		2		
	*Hypoponera*	8		8		
	*Leptogenys*	12		12	4	
	*Loboponera*	3		3		
	*Megaponera*	1	2	3		
	*Mesoponera*	4		4		
	*Odontomachus*	2		2		
	*Paltothyreus*	1	1	2		
	*Parvaponera*		1	1		
	*Phrynoponera*	3		3		
	*Platythyrea*	8		8		
	*Plectroctena*	7		7	1	
	*Psalidomyrmex*	3		3		
**Total**		76	4	80	6	
PROCERATIINAE						
	*Discothyrea*	4		4	1	
						
**Total**		4		4	1	
PSEUDOMYRMECINAE						
	*Tetraponera*	9		9		
**Total**		9		9		
**Global**		**545**	**191**	**736**	**156**	**4**

**Table 2.** Ant species cited for DRC, but discarded in the current list (NP = Not Present, DB = Dubious).

**Species**	**Status**	**Provinces**	**Reference**
*Bothroponeracrassa* (Emery, 1877)	DB	BC	Forel (1913c), Wheeler (1922b), Finzi (1939)
*Odontomachushaematodus* (Linnaeus, 1758)	NP	BC, BU, HU, KN, SU, TO	Forel (1909), Stitz (1910), Forel (1913a), Forel (1913c), Stitz (1916), Wheeler (1922a), Wheeler (1922b), Prins (1964)
*Oecophyllasmaragdina* (Fabricius, 1775)	NP	HL	Forel (1913a), Wheeler (1922b)
*Tetramoriumbicarinatum* (Nylander, 1846)	DB	-	Wetterer (2009)
*Tetramoriumdecem* Forel, 1913	NP	HU	Weber (1952b), rest of cited material re-identified as *T.venator* in Hita Garcia and Fisher (2014)
*Tetramoriumkelleri* Forel, 1887	NP	SK	Santschi (1924a)

**Table 3.** Ant species cited for DRC.

Subfamily	Genus	Species	Notes	Provinces	Reference
Apomyrminae	*Apomyrma*	*Apomyrmastygia* Brown, Gotwald & Levieux, 1971		KL	Boudinot (2015)
Dolichoderinae	*Axinidris*	*Axinidrisdenticulata* (Wheeler, 1922)		EQ, NK, TO	Wheeler (1922a), Wheeler (1922b), Shattuck (1991), Shattuck (1994), Snelling (2007)
Dolichoderinae	*Axinidris*	*Axinidrishypoclinoides* (Santschi, 1919)		TO	Santschi (1919c), Wheeler (1922b), Shattuck (1994), Bolton (2007), Snelling (2007)
Dolichoderinae	*Tapinoma*	*Tapinomaluridum* Emery, 1908		SA	Emery (1908), Wheeler (1922b), Shattuck (1994)
Dolichoderinae	*Tapinoma*	*Tapinomaluridumlongiceps* Wheeler, 1922		NU, SU	Wheeler (1922a), Wheeler (1922b), Baroni Urbani (1977), Shattuck (1994), Braet and Taylor (2008)
Dolichoderinae	*Tapinoma*	*Tapinomaschultzei* (Forel, 1910)		TO	Forel (1916), Wheeler (1922b), Shattuck (1994)
Dolichoderinae	*Technomyrmex*	*Technomyrmexalbipes* (Smith, 1861)		BC, KN	Santschi (1910), Wheeler (1922b)
Dolichoderinae	*Technomyrmex*	*Technomyrmexandrei* Emery, 1899		BU, HU, IT, KN, MA, NK, TO	Forel (1916), Stitz (1916), Wheeler (1922a), Wheeler (1922b), Santschi (1930a), Baroni Urbani (1977), Shattuck (1994), Bolton (2007), Van De Perre et al. (2018)
Dolichoderinae	*Technomyrmex*	*Technomyrmexcamerunensis* Emery, 1899		KN	Emery (1899)
Dolichoderinae	*Technomyrmex*	*Technomyrmexilgi* (Forel, 1910)		KL	Forel (1916), Wheeler (1922b)
Dolichoderinae	*Technomyrmex*	*Technomyrmexlasiops* Bolton, 2007		IT	Bolton (2007)
Dolichoderinae	*Technomyrmex*	*Technomyrmexlaurenti* (Emery, 1899)		BU, IT, MA, TO	Forel (1916), Wheeler (1922a), Wheeler (1922b), Shattuck (1994), Bolton (2007), Van De Perre et al. (2018)
Dolichoderinae	*Technomyrmex*	*Technomyrmexlujae* (Forel, 1905)		BU, EQ, KL, KS, SA, TO	Forel (1905), Forel (1911b), Forel (1916), Wheeler (1922a), Wheeler (1922b), Santschi (1926a), Santschi (1930a), Baroni Urbani (1977), Shattuck (1994), Bolton (2007), Van De Perre et al. (2018)
Dolichoderinae	*Technomyrmex*	*Technomyrmexmetandrei* Bolton, 2007		IT	Bolton (2007)
Dolichoderinae	*Technomyrmex*	*Technomyrmexmoerens* Santschi, 1913		BC, IT, KN, KS, MN, TO	Wheeler (1922b), Karavaiev (1926), Santschi (1930a), Weber (1943), Baroni Urbani (1977), Shattuck (1994), Bolton (2007), Van De Perre et al. (2018)
Dolichoderinae	*Technomyrmex*	*Technomyrmexnigriventris* Santschi, 1910		BC, KN, KS	Forel (1909), Wheeler (1922a), Wheeler (1922b), Santschi (1930a)
Dolichoderinae	*Technomyrmex*	*Technomyrmexpallipes* (Smith, 1876)		KS	Santschi (1930a), Baroni Urbani (1977), Shattuck (1994), Bolton (2007)
Dolichoderinae	*Technomyrmex*	*Technomyrmexparviflavus* Bolton, 2007		IT	Bolton (2007)
Dolichoderinae	*Technomyrmex*	*Technomyrmexrusticus* Santschi, 1930	Endemic	MN	Santschi (1930a), Baroni Urbani (1977), Shattuck (1994), Bolton (2007)
Dolichoderinae	*Technomyrmex*	*Technomyrmexschoutedeni* Forel, 1910		SA, TO	Forel (1910b), Emery (1913), Forel (1916), Wheeler (1922b), Baroni Urbani (1977), Shattuck (1994), Bolton (2007)
Dolichoderinae	*Technomyrmex*	*Technomyrmexsenex* Bolton, 2007		MA	Bolton (2007)
Dorylinae	*Aenictogiton*	*Aenictogitonattenuatus* Santschi, 1919	Endemic	HK, HL	Santschi (1919b), Wheeler (1922b), Santschi (1924a), Baroni Urbani (1977), Borowiec (2016)
Dorylinae	*Aenictogiton*	*Aenictogitonbequaerti* Forel, 1913	Endemic	HK, HL	Forel (1913a), Wheeler (1922b), Borowiec (2016)
Dorylinae	*Aenictogiton*	*Aenictogitonelongatus* Santschi, 1919	Endemic	BC	Santschi (1919b), Wheeler (1922b), Borowiec (2016)
Dorylinae	*Aenictogiton*	*Aenictogitonemeryi* Forel, 1913	Endemic	HK, TA	Forel (1913a), Wheeler (1922b), Santschi (1924a), Borowiec (2016)
Dorylinae	*Aenictogiton*	*Aenictogitonfossiceps* Emery, 1901		-	Emery (1901), Borowiec (2016)
Dorylinae	*Aenictogiton*	*Aenictogitonschoutedeni* Santschi, 1924	Endemic	KS	Santschi (1924a), Borowiec (2016)
Dorylinae	*Aenictogiton*	*Aenictogitonsulcatus* Santschi, 1919	Endemic	TA	Santschi (1919b), Wheeler (1922b), Baroni Urbani (1977), Borowiec (2016)
Dorylinae	*Aenictus*	*Aenictusalluaudifalcifer* Santschi, 1924	Endemic	IT, NK	Santschi (1924a), Borowiec (2016)
Dorylinae	*Aenictus*	*Aenictusbuttgenbachi* Forel, 1913		HK	Forel (1913a), Wheeler (1922b), Borowiec (2016)
Dorylinae	*Aenictus*	*Aenictuscongolensis* Santschi, 1911		BC	Baroni Urbani (1977)
Dorylinae	*Aenictus*	*Aenictuseugenii* Emery, 1895		BC, HK, NK, SK	Santschi (1924a), Gotwald and Cunningham-Van Someren (1976), Baroni Urbani (1977), Borowiec (2016), Gómez (2022)
Dorylinae	*Aenictus*	*Aenictusfuscovarius* Gerstäcker, 1859		HK	Forel (1913c), Wheeler (1922b), Santschi (1924a)
Dorylinae	*Aenictus*	*Aenictusmoebiisankisianus* Forel, 1913	Endemic	HK, HL	Forel (1913a), Borowiec (2016)
Dorylinae	*Aenictus*	*Aenictusraptor* Forel, 1913	Endemic	HK	Forel (1913a), Borowiec (2016)
Dorylinae	*Aenictus*	*Aenictussoudanicusbrunneus* Forel, 1913	Endemic	HK, HL	Forel (1913a), Borowiec (2016)
Dorylinae	*Aenictus*	*Aenictusweissi* Santschi, 1910		IT, NU	Santschi (1910), Wheeler (1922b), Baroni Urbani (1977), Borowiec (2016)Gómez (2022)
Dorylinae	*Dorylus*	*Dorylusacutus* Santschi, 1937	Endemic	-	Santschi (1939), Baroni Urbani (1977), Borowiec (2016)
Dorylinae	*Dorylus*	*Dorylusaffinis* Shuckard, 1840		BC, KN, SA	Forel (1909), Forel (1911b), Arnold (1915), Wheeler (1922b), Santschi (1923)
Dorylinae	*Dorylus*	*Dorylusaggressor* Santschi, 1923	Endemic	KC	Santschi (1923), Baroni Urbani (1977), Borowiec (2016)
Dorylinae	*Dorylus*	*Dorylusalluaudilobatus* Santschi, 1919	Endemic	EQ, LO	Santschi (1919b), Wheeler (1922b), Baroni Urbani (1977), Borowiec (2016)
Dorylinae	*Dorylus*	*Dorylusatratus* Smith, 1859		BC, TO	Wheeler (1922a), Wheeler (1922b), Santschi (1923)
Dorylinae	*Dorylus*	*Dorylusatriceps* Shuckard, 1840		BC, HU, TO	Forel (1909), Wheeler (1922a), Wheeler (1922b), Santschi (1935b)
Dorylinae	*Dorylus*	*Dorylusattenuatuslatinodis* Forel, 1920	Endemic	TO	Forel (1920), Wheeler (1922b), Borowiec (2016)
Dorylinae	*Dorylus*	*Dorylusattenuatus* Shuckard, 1840		BC, HK	Forel (1913a), Wheeler (1922b), Santschi (1939)
Dorylinae	*Dorylus*	*Dorylusbequaerti* Forel, 1913		BC, HK, HL, SU	Forel (1913a), Wheeler (1922a), Wheeler (1922b), Baroni Urbani (1977), Borowiec (2016)
Dorylinae	*Dorylus*	*Dorylusbraunsi* Emery, 1895		BC, KN	Emery (1895), Santschi (1910), Wheeler (1922b)
Dorylinae	*Dorylus*	*Dorylusbrevipennismarshalli* Emery, 1901		HU	Forel (1916), Wheeler (1922a), Wheeler (1922b)
Dorylinae	*Dorylus*	*Dorylusbrevipenniszimmermanni* Santschi, 1910		-	Borowiec (2016)
Dorylinae	*Dorylus*	*Dorylusbrevis* Santschi, 1919	Endemic	MN	Santschi (1919b), Wheeler (1922b), Borowiec (2016)
Dorylinae	*Dorylus*	*Doryluscongolensis* Santschi, 1910		BC, KN	Santschi (1910), Wheeler (1922a), Wheeler (1922b)
Dorylinae	*Dorylus*	*Dorylusconradti* Emery, 1895		HU	Wheeler (1922a), Wheeler (1922b)
Dorylinae	*Dorylus*	*Dorylusdepilisclarior* Santschi, 1917		SU	Santschi (1915), Wheeler (1922b), Borowiec (2016)
Dorylinae	*Dorylus*	*Dorylusdepilis* Emery, 1895		BC, BU, HK, HL, HU, KL, LU, MA, MO, SA, TO	Forel (1909), Forel (1911b), Forel (1913a), Forel (1913c), Wheeler (1922a), Wheeler (1922b), Santschi (1923)
Dorylinae	*Dorylus*	*Dorylusdepilisugandensis* Santschi, 1914		HU	Santschi (1923)
Dorylinae	*Dorylus*	*Dorylusemeryi* Mayr, 1896		-	Forel (1916), Wheeler (1922b)
Dorylinae	*Dorylus*	*Dorylusemeryiopacus* Forel, 1909		BC, HU	Forel (1909), Emery (1910), Forel (1913c), Forel (1916), Wheeler (1922a), Wheeler (1922b), Borowiec (2016)
Dorylinae	*Dorylus*	*Dorylusfimbriatus* (Shuckard, 1840)		HK, SA	Forel (1909), Wheeler (1922b)
Dorylinae	*Dorylus*	*Dorylusfulvusbadius* Gerstäcker, 1859		BC, KS, MA, NK, SA	Stitz (1911), Forel (1913a), Forel (1913c), Wheeler (1922b), Santschi (1935b), Prins (1963)
Dorylinae	*Dorylus*	*Dorylusfulvusdentifrons* Wasmann, 1904		IT, SA, TO	Wheeler (1922b), Santschi (1923), Santschi (1931), Baroni Urbani (1977), Borowiec (2016)
Dorylinae	*Dorylus*	*Dorylusfulvusobscurior* Wheeler, 1925		HU, SK, TO	Wheeler (1922a), Wheeler (1922b), Santschi (1935a)
Dorylinae	*Dorylus*	*Dorylusfunereusacherontus* Santschi, 1937		EQ	Santschi (1937b)
Dorylinae	*Dorylus*	*Dorylusfunereus* Emery, 1895		BC, EQ, HU, KS, MN, SA, TO	Forel (1909), Forel (1913a), Forel (1913c), Forel (1916), Wheeler (1922a), Wheeler (1922b), Santschi (1935a)
Dorylinae	*Dorylus*	*Dorylusfunereuspardus* Santschi, 1937	Endemic	TO	Santschi (1937b), Baroni Urbani (1977), Borowiec (2016)
Dorylinae	*Dorylus*	*Dorylusfunereusstygis* Santschi, 1937		HK	Santschi (1937b), Baroni Urbani (1977), Borowiec (2016)
Dorylinae	*Dorylus*	*Dorylusgaudens* Santschi, 1919	Endemic	IT	Santschi (1919b), Wheeler (1922b), Borowiec (2016)
Dorylinae	*Dorylus*	*Dorylusgribodoi* Emery, 1892		KS, SA	Forel (1913a), Wheeler (1922b)
Dorylinae	*Dorylus*	*Dorylushelvolus* (Linnaeus, 1764)		HK	Forel (1913a), Wheeler (1922b), Prins (1963)
Dorylinae	*Dorylus*	*Doryluskatanensis* Stitz, 1911	Endemic	NK	Wheeler (1922b), Borowiec (2016)
Dorylinae	*Dorylus*	*Doryluskohlichapini* Wheeler, 1922	Endemic	TO	Wheeler (1922a), Wheeler (1922b), Baroni Urbani (1977), Borowiec (2016)
Dorylinae	*Dorylus*	*Doryluskohlifrenisyi* Forel, 1916	Endemic	-	Forel (1916), Wheeler (1922b), Borowiec (2016)
Dorylinae	*Dorylus*	*Doryluskohliindocili*s Santschi, 1933	Endemic	MN	Santschi (1933), Baroni Urbani (1977), Borowiec (2016)
Dorylinae	*Dorylus*	*Doryluskohlilangi* Wheeler, 1922	Endemic	BC	Wheeler (1922a), Wheeler (1922b), Baroni Urbani (1977), Borowiec (2016)
Dorylinae	*Dorylus*	*Doryluskohlimilitaris* Santschi, 1923	Endemic	MO, TO	Santschi (1923), Baroni Urbani (1977), Borowiec (2016)
Dorylinae	*Dorylus*	*Doryluskohli* Wasmann, 1904		BC, BU, HU, SA, TO	Santschi (1921), Wheeler (1922a), Wheeler (1922b), Santschi (1933), van Boven (1968), Baroni Urbani (1977), Schöning et al. (2008), Borowiec (2016)
Dorylinae	*Dorylus*	*Dorylusmandibularispulchellus* Santschi, 1920		BC, KN, SA, TO	Wheeler (1922b)
Dorylinae	*Dorylus*	*Dorylusmoestusclaripennis* Santschi, 1919		HK, KG, KS	Santschi (1919b), Wheeler (1922b), Santschi (1923), Borowiec (2016)
Dorylinae	*Dorylus*	*Dorylusmoestus* Emery, 1895		BC, BU, KN, MO, SA, TO	Forel (1909), Forel (1913c), Stitz (1916), Wheeler (1922a), Santschi (1923), Borowiec (2016)
Dorylinae	*Dorylus*	*Dorylusniarembensis* (van Boven, 1972)	Endemic	IT	van Boven (1972), Borowiec (2016)
Dorylinae	*Dorylus*	*Dorylusnigricansarcens* (Westwood, 1847)		HU	Wheeler (1922a), Wheeler (1922b)
Dorylinae	*Dorylus*	*Dorylusnigricansburmeisteri* (Shuckard, 1840)		BC, EQ, SU, TO	Stitz (1916), Wheeler (1922a), Wheeler (1922b)
Dorylinae	*Dorylus*	*Dorylusnigricans* Illiger, 1802		BC, HK, KN, SU, TA	Forel (1909), Forel (1913a), Forel (1913c), Wheeler (1922b)
Dorylinae	*Dorylus*	*Dorylusnigricansmolestus* (Gerstäcker, 1859)		NK	Stitz (1911), Wheeler (1922b)
Dorylinae	*Dorylus*	*Dorylusnigricansrubellus* (Savage, 1849)		BC, TO	Forel (1911b), Forel (1913c), Wheeler (1922a), Wheeler (1922b)
Dorylinae	*Dorylus*	*Dorylusnigricanssjostedti* Emery, 1899		HU	Wheeler (1922a), Wheeler (1922b)
Dorylinae	*Dorylus*	*Dorylusnigricansterrificus* Santschi, 1923		SA, SK	Santschi (1923), van Boven (1967a), Baroni Urbani (1977), Borowiec (2016)
Dorylinae	*Dorylus*	*Doryluspolitus* Emery, 1901		BC, MN	Forel (1913c), Wheeler (1922b), Santschi (1933)
Dorylinae	*Dorylus*	*Dorylussavagei* Emery, 1895		BC	Forel (1909), Forel (1911b), Forel (1913c), Wheeler (1922b)
Dorylinae	*Dorylus*	*Dorylusschoutedeni* Santschi, 1923	Endemic	BC, KL, MN	Santschi (1923), Baroni Urbani (1977), Borowiec (2016)
Dorylinae	*Dorylus*	*Dorylusstadelmanni* Emery, 1895		-	Borowiec (2016)
Dorylinae	*Dorylus*	*Dorylusstanleyi* Forel, 1909		HK, IT	Forel (1909), Forel (1916), Wheeler (1922b), Santschi (1935b), Borowiec (2016)
Dorylinae	*Dorylus*	*Dorylusstaudinger*i Emery, 1895		HU	Wheeler (1922a), Wheeler (1922b), Borowiec (2016)
Dorylinae	*Dorylus*	*Dorylustermitarius* Wasmann, 1911	Endemic	TO	Wheeler (1922b), van Boven (1967b), Borowiec (2016)
Dorylinae	*Dorylus*	*Dorylustitan* Santschi, 1923		EQ, SA	Santschi (1923), Santschi (1933), Baroni Urbani (1977), Borowiec (2016)
Dorylinae	*Dorylus*	*Dorylustitanvinalli* Santschi, 1933	Endemic	EQ, MO	Santschi (1933), Baroni Urbani (1977), Borowiec (2016)
Dorylinae	*Dorylus*	*Doryluswilverthi* Emery, 1899		BC, BU, EQ, HU, IT, KN, KS, MO, NK, SA, SK, SU, TO	Emery (1899), Forel (1909), Stitz (1910), Forel (1913a), Forel (1913c), Forel (1916), Stitz (1916), Wheeler (1922a), Wheeler (1922b), Santschi (1935a), Wheeler (1943), Borowiec (2016)
Dorylinae	*Lioponera*	*Lioponeraforeli* (Santschi, 1914)		IT, MA, SK, TO	Wheeler (1922a), Wheeler (1922b), Brown (1975)
Dorylinae	*Lioponera*	*Lioponerankomoensis* (Forel, 1916)		TO	Forel (1916), Wheeler (1922b), Brown (1975), Borowiec (2016)
Dorylinae	*Parasyscia*	*Parasysciacenturio* (Brown, 1975)		NK, SK	Brown (1975), Borowiec (2016)
Dorylinae	*Parasyscia*	*Parasysciacribrinodis* Emery, 1899		HU	Wheeler (1922a), Wheeler (1922b)
Dorylinae	*Parasyscia*	*Parasyscianitidulus* (Brown, 1975)		TO	Weber (1949a), Borowiec (2016)
Dorylinae	*Simopone*	*Simoponeannettae* Kutter, 1976		TO	Bolton and Fisher (2012)
Dorylinae	*Simopone*	*Simoponebrunnea* Bolton & Fisher, 2012		IT	Bolton and Fisher (2012)
Dorylinae	*Simopone*	*Simoponeconradti* Emery, 1899		KN	Bolton and Fisher (2012)
Dorylinae	*Simopone*	*Simoponefulvinodis* Santschi, 1923	Endemic	BC	Santschi (1923), Bolton and Fisher (2012), Borowiec (2016)
Dorylinae	*Simopone*	*Simoponegrandis* Santschi, 1923		KS, MN	Santschi (1923), Brown (1975), Bolton and Fisher (2012), Borowiec (2016)
Dorylinae	*Simopone*	*Simoponeschoutedeni* Santschi, 1923		KS, TO	Santschi (1923), Brown (1975), Bolton and Fisher (2012), Borowiec (2016)
Dorylinae	*Simopone*	*Simoponewilburi* Weber, 1949		IT, NK	Weber (1949a), Bolton and Fisher (2012), Borowiec (2016)
Dorylinae	*Vicinopone*	*Vicinoponeconciliatrix* (Bronw, 1975)		TO	Brown (1975), Bolton and Fisher (2012)
Dorylinae	*Zasphinctus*	*Zasphinctussarowiwai* Hita Garcia, 2017		IT	Hita Garcia et al. (2017b)
Formicinae	*Anoplolepis*	*Anoplolepiscarinata* (Emery, 1899)		BC, HU, TO	Forel (1913c), Wheeler (1922b), Santschi (1930a), Santschi (1935a)
Formicinae	*Anoplolepis*	*Anoplolepiscustodiens* (Smith, 1858)		BC	Emery (1899), Forel (1909), Wheeler (1922a), Wheeler (1922b), Prins (1963)
Formicinae	*Anoplolepis*	*Anoplolepisfallax* (Mayr, 1865)		MO	Forel (1909), Wheeler (1922b)
Formicinae	*Anoplolepis*	*Anoplolepiskohli* (Forel, 1916)		TO	Forel (1916), Wheeler (1922b), Lapolla and Fisher (2014)
Formicinae	*Anoplolepis*	*Anoplolepistenella* (Santschi, 1911)		BC, BU, HU, TO	Forel (1909), Santschi (1911), Wheeler (1922a), Wheeler (1922b)
Formicinae	*Aphomomyrmex*	*Aphomomyrmexafer* Emery, 1899		BC, SA	Snelling (1979)
Formicinae	*Camponotus*	*Camponotusaberrans* Mayr, 1895		TA	Santschi (1915), Wheeler (1922b)
Formicinae	*Camponotus*	*Camponotusacvapimensis* Mayr, 1862		BC, BU, EQ, HU, KN, LU, MN, SU, TO, TU	Forel (1911b), Forel (1913a), Forel (1913c), Forel (1915), Forel (1916), Wheeler (1922a), Wheeler (1922b), Santschi (1935a)
Formicinae	*Camponotus*	*Camponotusaequatorialiskohli* Forel, 1915	Endemic	TO	Forel (1915), Wheeler (1922b)
Formicinae	*Camponotus*	*Camponotusaequatorialis* Roger, 1863		TO	Van De Perre et al. (2018)
Formicinae	*Camponotus*	*Camponotusargus* Santschi, 1935	Endemic	MO	Santschi (1935a)
Formicinae	*Camponotus*	*Camponotusatriscapus* Santschi, 1926	Endemic	EQ	Santschi (1926b)
Formicinae	*Camponotus*	*Camponotusauropubens* Forel, 1894		TO	Wheeler (1922a), Wheeler (1922b)
Formicinae	*Camponotus*	*Camponotusbarbarossamicipsa* Wheeler, 1922		KN, MN	Wheeler (1922a), Wheeler (1922b)
Formicinae	*Camponotus*	*Camponotusbarbarossasulcatinasis* Santschi, 1926	Endemic	BC, HU, IT	Santschi (1926b)
Formicinae	*Camponotus*	*Camponotusbayeri* Forel, 1913		HU, TA	Forel (1913c), Wheeler (1922a), Wheeler (1922b), Weber (1943)
Formicinae	*Camponotus*	*Camponotusbraunsi* Mayr, 1895		KN	Forel (1913c), Wheeler (1922b)
Formicinae	*Camponotus*	*Camponotusbrutus* Forel, 1886		BC, BU, EQ, HL, HU, IT, KN, KS, MO, NK, SA, SU, TO	Forel (1909), Forel (1911b), Forel (1913a), Forel (1913c), Forel (1916), Stitz (1916), Wheeler (1922a), Wheeler (1922b), Wheeler (1925), Santschi (1935a), Weber (1943), Weber (1964)
Formicinae	*Camponotus*	*Camponotusbuchneri* Forel, 1886		EQ, HU, KS, MA, TO	Wheeler (1922a), Wheeler (1922b), Santschi (1923)
Formicinae	*Camponotus*	*Camponotusburgeoni* Santschi, 1926	Endemic	HU, KS	Santschi (1926b)
Formicinae	*Camponotus*	*Camponotuscaesar* Forel, 1886		HK, HU	Wheeler (1922a), Wheeler (1922b)
Formicinae	*Camponotus*	Camponotuscaesar imperator Emery, 1899		MA, TO	Wheeler (1922a), Wheeler (1922b)
Formicinae	*Camponotus*	*Camponotuscarbo* Emery, 1877		-	Levieux (1972)
Formicinae	*Camponotus*	*Camponotuschapiniganzii* Weber, 1943		-	Weber (1943)
Formicinae	*Camponotus*	*Camponotuschapini* Wheeler, 1922		HU	Wheeler (1922a), Wheeler (1922b), Levieux (1972)
Formicinae	*Camponotus*	*Camponotuschrysurusacutisquamis* Mayr, 1902		BC	Forel (1913a), Forel (1916), Wheeler (1922b)
Formicinae	*Camponotus*	*Camponotuschrysurusapellis* Santschi, 1911		BC, SU	Forel (1911b), Stitz (1916), Wheeler (1922b)
Formicinae	*Camponotus*	*Camponotuschrysurus* Gerstäcker, 1871		BC, HK, HL, KN, TO, TU	Forel (1913a), Forel (1913c), Forel (1915), Van De Perre et al. (2018)
Formicinae	*Camponotus*	*Camponotuschrysurusyvonnae* Forel, 1920	Endemic	TO	Forel (1920), Wheeler (1922b)
Formicinae	*Camponotus*	*Camponotuscinctellus* (Gerstäcker, 1859)		EQ, KN, MO, SU, TU	Forel (1913a), Wheeler (1922a), Wheeler (1922b)
Formicinae	*Camponotus*	*Camponotusconfluensbequaerti* Forel, 1913		HL	Forel (1913a), Wheeler (1922b)
Formicinae	*Camponotus*	*Camponotusconfluens* Forel, 1913	Endemic	HK, HL	Forel (1913a), Wheeler (1922b)
Formicinae	*Camponotus*	*Camponotuscongolensis* Emery, 1899		BC, HK, HU, KN, NK	Emery (1899), Forel (1909), Forel (1913a), Wheeler (1922a), Wheeler (1922b), Menozzi (1932)
Formicinae	*Camponotus*	*Camponotuscongolensisweissi* Santschi, 1911		BC	Forel (1913a), Wheeler (1922b)
Formicinae	*Camponotus*	*Camponotuscosmicus* (Smith, 1858)		SU	Stitz (1916), Wheeler (1922b)
Formicinae	*Camponotus*	*Camponotusetiolipes* Bolton, 1995		HL, IT, NK	Stitz (1911), Forel (1913a), Wheeler (1922b), Weber (1943)
Formicinae	*Camponotus*	*Camponotuseugeniaeamplior* Forel, 1913	Endemic	HL, MA	Forel (1913a), Forel (1913c), Wheeler (1922b)
Formicinae	*Camponotus*	*Camponotusferreriakka* Forel, 1916	Endemic	-	Forel (1916), Wheeler (1922b)
Formicinae	*Camponotus*	*Camponotusflavomarginatus* Mayr, 1862		BC, BU, HU, SA	Wheeler (1922a), Wheeler (1922b), Santschi (1935a)
Formicinae	*Camponotus*	*Camponotusflorius* Santschi, 1926		BC	Santschi (1933)
Formicinae	*Camponotus*	*Camponotusforaminosus* Forel, 1879		HU, KN, TO	Stitz (1910), Forel (1915), Forel (1916), Wheeler (1922a), Wheeler (1922b), Van De Perre et al. (2018)
Formicinae	*Camponotus*	*Camponotusfulvopilosus* (De Geer, 1778)		HK	Wheeler (1922b)
Formicinae	*Camponotus*	*Camponotusfurvus* Santschi, 1911		KS	Santschi (1933)
Formicinae	*Camponotus*	*Camponotusguttatus* Emery, 1899		SU	Wheeler (1922a), Wheeler (1922b)
Formicinae	*Camponotus*	*Camponotushaereticus* Santschi, 1914		EQ	Wheeler (1922a), Wheeler (1922b)
Formicinae	*Camponotus*	*Camponotuslangijejunus* Santschi, 1926	Endemic	TA	Santschi (1926a)
Formicinae	*Camponotus*	*Camponotuslangi* Wheeler, 1922		HU	Wheeler (1922a), Wheeler (1922b)
Formicinae	*Camponotus*	*Camponotuslilianaecornutus* Forel, 1913	Endemic	HK	Forel (1913a), Wheeler (1922b)
Formicinae	*Camponotus*	*Camponotuslilianae* Forel, 1913		HL	Forel (1913a), Wheeler (1922b)
Formicinae	*Camponotus*	*Camponotuslongipalpis* Santschi, 1926	Endemic	KS	Santschi (1926b)
Formicinae	*Camponotus*	*Camponotusmaculatus* (Fabricius, 1782)		BU, HK, HU, NK, SU, TA	Forel (1911b), Forel (1913a), Forel (1913c), Santschi (1915), Stitz (1916), Emery (1920), Wheeler (1922a), Wheeler (1922b), Santschi (1930a), Weber (1943), Prins (1963)
Formicinae	*Camponotus*	*Camponotusmaguassa* Wheeler, 1922	Endemic	TO	Wheeler (1922a), Wheeler (1922b)
Formicinae	*Camponotus*	*Camponotusmassinissa* Wheeler, 1922	Endemic	HU	Wheeler (1922a), Wheeler (1922b)
Formicinae	*Camponotus*	*Camponotusmaynei* Forel, 1916		-	Forel (1916), Wheeler (1922b), Braet and Taylor (2008)
Formicinae	*Camponotus*	*Camponotusmayri* Forel, 1879		NK	Wheeler (1922b), Prins (1964)
Formicinae	*Camponotus*	*Camponotusoculatior* Santschi, 1935	Endemic	KN	Santschi (1935a)
Formicinae	*Camponotus*	*Camponotusolivieridelagoensis* Forel, 1894		BC	Forel (1909)
Formicinae	*Camponotus*	*Camponotusolivieri* Forel, 1886		KN	Forel (1913c), Wheeler (1922b)
Formicinae	*Camponotus*	*Camponotusolivierilemma* Forel, 1886		BC, KN, TU	Forel (1913c), Wheeler (1922b)
Formicinae	*Camponotus*	*Camponotusolivieriosiris* Forel, 1911	Endemic	KN, MO	Forel (1911b), Santschi (1915), Wheeler (1922b), Santschi (1935a)
Formicinae	*Camponotus*	*Camponotusolivierisorptus* Santschi, 1915	Endemic	BC, EQ, KN, MN, TO	Wheeler (1922a), Wheeler (1922b)
Formicinae	*Camponotus*	*Camponotusparadoxus* (Mayr, 1866)	Endemic	TO	Van De Perre et al. (2018)
Formicinae	*Camponotus*	*Camponotusperrisiidensipunctatus* Stitz, 1916		SU	Stitz (1916), Wheeler (1922b)
Formicinae	*Camponotus*	*Camponotusperrisiijucundus* Santschi, 1911		BC, HU, KN, MN, NU, SU	Santschi (1911), Santschi (1913b), Stitz (1916), Wheeler (1922a), Wheeler (1922b), Wheeler (1936)
Formicinae	*Camponotus*	*Camponotusperrisiinigeriensis* Santschi, 1914		TA	Santschi (1915), Wheeler (1922b)
Formicinae	*Camponotus*	*Camponotuspolyrhachioides* Emery, 1898		BC, EQ, MN, MO	Forel (1913c), Wheeler (1922a), Wheeler (1922b), Santschi (1923)
Formicinae	*Camponotus*	*Camponotuspompeiuscassius* Wheeler, 1922		HU, TO	Wheeler (1922a), Wheeler (1922b), Weber (1943)
Formicinae	*Camponotus*	*Camponotuspompeius* Forel, 1886		SU	Stitz (1916), Wheeler (1922b)
Formicinae	*Camponotus*	*Camponotuspompeiusiota* Santschi, 1926	Endemic	BU	Santschi (1926a)
Formicinae	*Camponotus*	*Camponotuspompeiusmarius* Emery, 1899		BU, HU, KN, SU	Stitz (1916), Wheeler (1922a), Wheeler (1922b), Santschi (1926a)
Formicinae	*Camponotus*	*Camponotusposticus* Santschi, 1926		TA	Santschi (1926a)
Formicinae	*Camponotus*	*Camponotusprosulcatus* Santschi, 1935	Endemic	MN	Santschi (1935a)
Formicinae	*Camponotus*	*Camponotuspuberulus* Emery, 1897		IT, KN	Forel (1911b), Forel (1913a), Wheeler (1922b)
Formicinae	*Camponotus*	*Camponotusrotundinodis* Santschi, 1935	Endemic	MO	Santschi (1935a), Weber (1943)
Formicinae	*Camponotus*	*Camponotusroubaudi* Santschi, 1911		NK	Forel (1913a), Wheeler (1922b)
Formicinae	*Camponotus*	*Camponotusrufoglaucuscontroversus* Santschi, 1916		HK	Santschi (1916), Wheeler (1922b), Santschi (1926a), Santschi (1935a)
Formicinae	*Camponotus*	*Camponotusrufoglaucussyphax* Wheeler, 1922		BC, SU	Wheeler (1922a), Wheeler (1922b)
Formicinae	*Camponotus*	*Camponotussankisianus* Forel, 1913		BC, HK, HL	Forel (1913a), Santschi (1913b), Wheeler (1922b), Arnold (1946)
Formicinae	*Camponotus*	*Camponotusschoutedeni* Forel, 1911		BC, KN, MN, MO, NK	Forel (1911b), Forel (1913a), Forel (1913c), Wheeler (1922b), Santschi (1935a)
Formicinae	*Camponotus*	*Camponotussericeus* (Fabricius, 1798)		BU, HK, HU, NK, SK, SU	Stitz (1916), Wheeler (1922a), Wheeler (1922b), Prins (1963), Prins (1964), Weber (1964)
Formicinae	*Camponotus*	*Camponotussericeussulgeri* Santschi, 1913		SU	Stitz (1916), Wheeler (1922b)
Formicinae	*Camponotus*	*Camponotussimus* Emery, 1908		SA	Emery (1908), Wheeler (1922b)
Formicinae	*Camponotus*	*Camponotussimusmanidis* Forel, 1909	Endemic	BC	Forel (1909), Forel (1911b), Wheeler (1922b)
Formicinae	*Camponotus*	*Camponotussolon* Forel, 1886		BC, BU, HK, HU, KN, KS, MA, SA, SU, TO	Forel (1909), Forel (1910b), Stitz (1910), Forel (1913c), Forel (1915), Forel (1916), Wheeler (1922a), Wheeler (1922b)
Formicinae	*Camponotus*	*Camponotussolonjugurtha* Emery, 1925	Endemic	TO	Wheeler (1922a), Wheeler (1922b)
Formicinae	*Camponotus*	*Camponotusvestitus* (Smith, 1858)		HK, TA	Forel (1913c), Wheeler (1922b)
Formicinae	*Camponotus*	*Camponotusvestitusanthracinus* Santschi, 1930		SA	Santschi (1935a)
Formicinae	*Camponotus*	*Camponotusvestitusintuens* Santschi, 1926		IT, KN	Forel (1913a), Wheeler (1922b), Santschi (1935a)
Formicinae	*Camponotus*	*Camponotusvestituslujai* Santschi, 1930		SA	Santschi (1930a)
Formicinae	*Camponotus*	*Camponotusvividus* (Smith, 1858)		BC, EQ, HK, KN, SA, SU, TO	Wheeler (1922a), Wheeler (1922b), Weber (1943), Weber (1964), Van De Perre et al. (2018)
Formicinae	*Camponotus*	*Camponotusvividuscato* Forel, 1913		BC, BU, HL, HU, MN, TO	Forel (1913a), Wheeler (1922a), Wheeler (1922b), Santschi (1926c), Santschi (1935a), Weber (1943)
Formicinae	*Camponotus*	*Camponotusvividusmeinerti* Forel, 1886		BC, EQ, KN, KS, SA, SU	Santschi (1910), Forel (1911b), Forel (1913a), Forel (1913c), Forel (1916), Stitz (1916), Santschi (1926c), Santschi (1935a)
Formicinae	*Camponotus*	*Camponotusvividusreginae* Forel, 1901		HK, SA	Forel (1911b), Forel (1913a), Wheeler (1922b)
Formicinae	*Camponotus*	*Camponotusvividussemidepilis* Wheeler, 1922		BC, HU, KN	Wheeler (1922a), Wheeler (1922b), Santschi (1926c)
Formicinae	*Camponotus*	*Camponotusvulpus* Santschi, 1926	Endemic	MN	Santschi (1926b)
Formicinae	*Camponotus*	*Camponotuswellmani* Forel, 1909		HK, HU, NK, TA, TO	Forel (1909), Forel (1913a), Forel (1916), Wheeler (1922a), Wheeler (1922b)
Formicinae	*Camponotus*	*Camponotuswellmanigamma* Santschi, 1926		KL	Santschi (1926a)
Formicinae	*Camponotus*	*Camponotuswellmanirufipartis* Forel, 1916	Endemic	HU	Forel (1916), Wheeler (1922b)
Formicinae	*Lepisiota*	*Lepisiotaalbata* (Santschi, 1935)	Endemic	KS	Santschi (1935a)
Formicinae	*Lepisiota*	*Lepisiotaambigua* (Santschi, 1935)		HU	Santschi (1935a)
Formicinae	*Lepisiota*	*Lepisiotacacozela* (Stitz, 1916)		HU, MO	Wheeler (1922a), Wheeler (1922b), Santschi (1935a)
Formicinae	*Lepisiota*	*Lepisiotacanescens* (Emery, 1897)		BC, KN, SK, TO	Forel (1911b), Stitz (1911), Wheeler (1922a), Wheeler (1922b)
Formicinae	*Lepisiota*	*Lepisiotacanescenslatior* (Santschi, 1935)		HU	Santschi (1935a)
Formicinae	*Lepisiota*	*Lepisiotacapensis* (Mayr, 1862)		HK, HL	Forel (1913a), Wheeler (1922b)
Formicinae	*Lepisiota*	*Lepisiotacapensisanceps* (Forel, 1916)		HU, TO	Forel (1916), Wheeler (1922a), Wheeler (1922b), Weber (1943)
Formicinae	*Lepisiota*	*Lepisiotacapensisguineensis* (Mayr, 1902)		BC	Wheeler (1922a), Wheeler (1922b)
Formicinae	*Lepisiota*	*Lepisiotacapensissimplicoides* (Forel, 1907)		BC, HL	Wheeler (1922b)
Formicinae	*Lepisiota*	*Lepisiotacapensisspecularis* (Santschi, 1935)		HU, SU	Santschi (1935a)
Formicinae	*Lepisiota*	*Lepisiotacapitata* (Forel, 1913)	Endemic	HL	Forel (1913a), Wheeler (1922b)
Formicinae	*Lepisiota*	*Lepisiotacarbonaria* (Emery, 1892)		BC, IT, SK, TO	Stitz (1911), Wheeler (1922a), Wheeler (1922b), Finzi (1939)
Formicinae	*Lepisiota*	*Lepisiotacurta* (Emery, 1897)		MO	Forel (1913c), Wheeler (1922b), Finzi (1939)
Formicinae	*Lepisiota*	*Lepisiotagerardi* (Santschi, 1915)	Endemic	TA	Santschi (1915), Wheeler (1922b)
Formicinae	*Lepisiota*	*Lepisiotahirsuta* (Santschi, 1914)		KS	Santschi (1935a)
Formicinae	*Lepisiota*	*Lepisiotahirsutasetosella* (Santschi, 1935)	Endemic	BC, KN, MN	Santschi (1935a)
Formicinae	*Lepisiota*	*Lepisiotaimperfectacongolensis* (Santschi, 1935)	Endemic	MO	Santschi (1935a)
Formicinae	*Lepisiota*	*Lepisiotaincisa* (Forel, 1913)		HK	Forel (1913a), Wheeler (1922b), Santschi (1935a), Prins (1964)
Formicinae	*Lepisiota*	*Lepisiotanigrisetosa* (Santschi, 1935)	Endemic	KS	Santschi (1935a)
Formicinae	*Lepisiota*	*Lepisiotaoculata* (Santschi, 1935)		HU	Santschi (1935a), Braet and Taylor (2008)
Formicinae	*Lepisiota*	*Lepisiotapalpalis* (Santschi, 1935)	Endemic	BC	Santschi (1935a)
Formicinae	*Lepisiota*	*Lepisiotapiliscapa* (Santschi, 1935)	Endemic	MN	Santschi (1935a)
Formicinae	*Lepisiota*	*Lepisiotapiliscapalongipilosa* (Santschi, 1935)	Endemic	TA	Santschi (1935a)
Formicinae	*Lepisiota*	*Lepisiotapiliscapapunctifrons* (Santschi, 1935)	Endemic	MO	Santschi (1935a)
Formicinae	*Lepisiota*	*Lepisiotaschoutedeni* (Santschi, 1935)	Endemic	HU	Santschi (1935a)
Formicinae	*Lepisiota*	*Lepisiotasubmetallica* (Arnold, 1920)		KS	Santschi (1935a)
Formicinae	*Lepisiota*	*Lepisiotatenuipilis* (Santschi, 1935)	Endemic	BC	Santschi (1935a)
Formicinae	*Lepisiota*	*Lepisiotavalidiuscula* (Emery, 1897)		BC, HL	Forel (1913a), Wheeler (1922a), Wheeler (1922b), Weber (1943), Santschi (1935a)
Formicinae	*Nylanderia*	*Nylanderialepida* (Santschi, 1915)		TO	Forel (1916), Wheeler (1922b), Lapolla et al. (2011)
Formicinae	*Nylanderia*	*Nylanderiamendica* (Menozzi, 1942)		IT	Lapolla et al. (2011)
Formicinae	*Nylanderia*	*Nylanderiavividula* (Nylander, 1846)		BU	Wheeler (1922a)
Formicinae	*Nylanderia*	*Nylanderiawaelbroecki* (Emery, 1899)	Endemic	KN	Emery (1899), Forel (1909), Forel (1911b), Wheeler (1922b), Lapolla et al. (2011)
Formicinae	*Oecophylla*	*Oecophyllalonginoda* (Latreille, 1802)		BC, BU, EQ, HL, HU, IT, KC, KL, KN, KS, MA, MN, MO, NK, SA, SK, SU, TO, TU	Forel (1909), Santschi (1910), Forel (1913a), Forel (1913c), Stitz (1916), Wheeler (1922a), Wheeler (1922b), Santschi (1935a), Weber (1949a), Wetterer (2017a), Wetterer (2017b), Van De Perre et al. (2018)
Formicinae	*Oecophylla*	*Oecophyllalonginodaannectens* Wheeler, 1922		BC, HU, TO	Wheeler (1922a), Wheeler (1922b), Cole and Jones (1948)
Formicinae	*Oecophylla*	*Oecophyllalonginodafusca* Emery, 1899		HU, SU, TO	Stitz (1916), Wheeler (1922a), Wheeler (1922b), Cole and Jones (1948)
Formicinae	*Oecophylla*	*Oecophyllalonginodarubriceps* Wheeler, 1922		BC, BU, TO	Santschi (1919a), Wheeler (1922a), Wheeler (1922b), Cole and Jones (1948)
Formicinae	*Oecophylla*	*Oecophyllalonginodarufescens* Santschi, 1928		HU, MO	Santschi (1928b)
Formicinae	*Oecophylla*	*Oecophyllalonginodataeniata* Santschi, 1928	Endemic	KL	Santschi (1928b)
Formicinae	*Paraparatrechina*	*Paraparatrechinabufona* (Wheeler, 1922)		HU	Wheeler (1922a), Wheeler (1922b), Weber and Anderson (1950), Lapolla (2004)
Formicinae	*Paraparatrechina*	*Paraparatrechinasubtilis* (Santschi, 1920)		IT	Lapolla et al. (2010)
Formicinae	*Paraparatrechina*	*Paraparatrechinaweissi* (Santschi, 1910)		BU, HU, IT	Wheeler (1922a), Wheeler (1922b), Weber and Anderson (1950), Brown (1957b), Lapolla (2004)
Formicinae	*Paratrechina*	*Paratrechinalongicornis* (Latreille, 1802)	Exotic	BC, IT, KN, KS, MN, SU, TO	Forel (1901b), Forel (1913a), Forel (1913c), Forel (1916), Stitz (1916), Wheeler (1922a), Wheeler (1922b), Wetterer (2008b)
Formicinae	*Plagiolepis*	*Plagiolepisexigua* Forel, 1894	Endemic	HL	Forel (1913a), Forel (1916), Wheeler (1922b), Finzi (1939)
Formicinae	*Plagiolepis*	*Plagiolepismediorufa* Forel, 1916		TO	Forel (1916), Wheeler (1922a), Wheeler (1922b)
Formicinae	*Plagiolepis*	*Plagiolepispuncta* Forel, 1910		TO	Forel (1916), Wheeler (1922b)
Formicinae	*Polyrhachis*	*Polyrhachisaerope* Wheeler, 1922		HU	Wheeler (1922a), Wheeler (1922b), Bolton (1973)
Formicinae	*Polyrhachis*	*Polyrhachisalexisi* Forel, 1916		-	Forel (1916), Wheeler (1922b), Bolton (1973), Rigato (2016)
Formicinae	*Polyrhachis*	*Polyrhachisalluaudi* Emery, 1892		TO	Forel (1916), Wheeler (1922a), Wheeler (1922b), Bolton (1973)
Formicinae	*Polyrhachis*	*Polyrhachisconcava* André, 1889		BC, BU, HU, IT, KL, KN, MN, TO, TU	Forel (1909), Forel (1911b), Forel (1913c), Forel (1916), Wheeler (1922a), Wheeler (1922b), Bolton (1973)
Formicinae	*Polyrhachis*	*Polyrhachiscornuta* Stitz, 1910	Endemic	KN	Stitz (1910), Wheeler (1922b), Bolton (1973)
Formicinae	*Polyrhachis*	*Polyrhachiscurta* André, 1890		KN	Wheeler (1922b), Bolton (1973)
Formicinae	*Polyrhachis*	*Polyrhachisdecemdentata* André, 1889		BC, EQ, HU, KN, KS, MO, TO	Forel (1911b), Forel (1915), Forel (1916), Wheeler (1922a), Wheeler (1922b), Santschi (1923), Bolton (1973)
Formicinae	*Polyrhachis*	*Polyrhachisepinotalis* Santschi, 1924		BC, HK, NK, SK	Forel (1913c), Wheeler (1922b), Rigato (2016)
Formicinae	*Polyrhachis*	*Polyrhachisesarata* Bolton, 1973		BC	Rigato (2016)
Formicinae	*Polyrhachis*	*Polyrhachisfissa* Mayr, 1902		BC, KS, MA, MN	Wheeler (1922a), Wheeler (1922b), Santschi (1923), Bolton (1973)
Formicinae	*Polyrhachis*	*Polyrhachisgagates* Smith, 1858		BC, HL, KG, KL, KN, LO, MA, MN, NK, SU, TA	Forel (1909), Santschi (1910), Forel (1910b), Forel (1913a), Forel (1913b), Forel (1913c), Wheeler (1922a), Wheeler (1922b), Prins (1963), Prins (1964), Bolton (1973)
Formicinae	*Polyrhachis*	*Polyrhachiskohli* Forel, 1916	Endemic	-	Forel (1916), Wheeler (1922b), Rigato (2016)
Formicinae	*Polyrhachis*	*Polyrhachislaboriosa* Smith, 1858		BC, EQ, HU, KN, KS, LO, LU, MN, MO, SA, TO, TU	Forel (1911b), Forel (1913a), Forel (1915), Forel (1916), Wheeler (1922a), Wheeler (1922b), Santschi (1924a), Bolton (1973)
Formicinae	*Polyrhachis*	*Polyrhachislatispina* Emery, 1925		EQ, HU, TO	Wheeler (1922a), Wheeler (1922b), Bolton (1973)
Formicinae	*Polyrhachis*	*Polyrhachislauta* Santschi, 1910		KN	Forel (1913c), Wheeler (1922b), Bolton (1973)
Formicinae	*Polyrhachis*	*Polyrhachislimitis* Santschi, 1939		MA	Bolton (1973), Rigato (2016)
Formicinae	*Polyrhachis*	*Polyrhachismilitaris* (Fabricius, 1782)		BC, BU, EQ, HK, HL, HU, IT, KC, KL, KN, LO, LU, MA, MN, MO, NK, SA, SK, SU, TA, TO, TU	Forel (1909), Stitz (1911), Forel (1913a), Forel (1913c), Forel (1916), Stitz (1916), Wheeler (1922a), Wheeler (1922b), Santschi (1924a), Wheeler (1925), Bernard (1953), Bolton (1973), Rigato (2016), Van De Perre et al. (2018)
Formicinae	*Polyrhachis*	*Polyrhachismonista* Santschi, 1910		TO	Forel (1916), Wheeler (1922b), Bolton (1973)
Formicinae	*Polyrhachis*	*Polyrhachisnigrita* Mayr, 1895		BU, MO	Santschi (1919b), Wheeler (1922a), Wheeler (1922b), Bolton (1973)
Formicinae	*Polyrhachis*	*Polyrhachisotleti* Forel, 1916		TO	Forel (1916), Wheeler (1922b), Bolton (1973), Rigato (2016)
Formicinae	*Polyrhachis*	*Polyrhachisrevoili* André, 1887		BC, KS, SA	Forel (1913a), Forel (1916), Wheeler (1922a), Wheeler (1922b)
Formicinae	*Polyrhachis*	*Polyrhachisrufipalpis* Santschi, 1910		BC, KS, MN	Forel (1913c), Wheeler (1922b), Santschi (1923), Bolton (1973)
Formicinae	*Polyrhachis*	*Polyrhachisschistacea* (Gerstäcker, 1859)		BC, BU, HK, HL, HU, IT, KC, KL, KN, KS, LO, LU, MA, MN, NK, NU, SA, SK, SU, TA, TO	Forel (1910b), Santschi (1910), Stitz (1910), Forel (1913a), Forel (1913c), Santschi (1913b), Santschi (1914), Stitz (1916), Wheeler (1922a), Wheeler (1922b), Santschi (1923), Santschi (1933), Weber (1943), Prins (1963), Prins (1964), Bolton (1973)
Formicinae	*Polyrhachis*	*Polyrhachisspinicola* Forel, 1894		-	Prins (1964)
Formicinae	*Polyrhachis*	*Polyrhachisspitteleri* Forel, 1916		-	Forel (1916), Wheeler (1922b), Bolton (1973), Rigato (2016)
Formicinae	*Polyrhachis*	*Polyrhachissulcata* André, 1895		-	Wheeler (1922b)
Formicinae	*Polyrhachis*	*Polyrhachisviscosa* Smith, 1858		HU, KL, MN	Wheeler (1922a), Wheeler (1922b), Santschi (1923), Bolton (1973), Rigato (2016)
Formicinae	*Polyrhachis*	*Polyrhachisvolkarti* Forel, 1916		-	Forel (1916), Wheeler (1922b), Bolton (1973), Rigato (2016)
Formicinae	*Polyrhachis*	*Polyrhachisweissi* Santschi, 1910		HU, SA, TO	Forel (1915), Wheeler (1922b), Bolton (1973), Rigato (2016), Van De Perre et al. (2018)
Formicinae	*Polyrhachis*	*Polyrhachiswellmani* Forel, 1909		HK	Bolton (1973)
Formicinae	*Santschiella*	*Santschiellakohli* Forel, 1916		TO	Forel (1916), Wheeler (1922b)
Myrmicinae	*Atopomyrmex*	*Atopomyrmexcryptoceroides* Emery, 1892		BC, EQ, HK, IT, KS, SA, SU, TO	Forel (1911a), Forel (1913a), Forel (1915), Forel (1916), Wheeler (1922a), Wheeler (1922b), Bolton (1981a), Van De Perre et al. (2018)
Myrmicinae	*Atopomyrmex*	*Atopomyrmexmocquerysi* André, 1889		BC, BU, EQ, HL, HU, MN, MO, SA, TO	Forel (1911b), Forel (1913a), Wheeler (1922a), Wheeler (1922b), Santschi (1923), Santschi (1924a), Bolton (1981a), Van De Perre et al. (2018)
Myrmicinae	*Bondroitia*	*Bondroitialujae* (Forel, 1909)		KC, SA	Forel (1909), Wheeler (1922b), Santschi (1923), Bolton (1987)
Myrmicinae	*Calyptomyrmex*	*Calyptomyrmexbrevis* Weber, 1943		IT, NK	Weber (1952b), Bolton (1981b)
Myrmicinae	*Calyptomyrmex*	*Calyptomyrmexclavatus* Weber, 1952		TO	Bolton (1981b)
Myrmicinae	*Calyptomyrmex*	*Calyptomyrmexduhun* Bolton, 1981		NK	Bolton (1981b)
Myrmicinae	*Calyptomyrmex*	*Calyptomyrmexnummuliticus* Santschi, 1914		IT, NK	Weber (1952b), Bolton (1981b)
Myrmicinae	*Calyptomyrmex*	*Calyptomyrmexpiripilis* Santschi, 1923		IT, NK, SU, TA	Santschi (1923), Bolton (1981b)
Myrmicinae	*Calyptomyrmex*	*Calyptomyrmexrennefer* Bolton, 1981		NK, SK	Bolton (1981b)
Myrmicinae	*Calyptomyrmex*	*Calyptomyrmexshasu* Bolton, 1981		NK	Bolton (1981b)
Myrmicinae	*Cardiocondyla*	*Cardiocondylaemeryi* Forel, 1881		BC, KN	Forel (1913c), Wheeler (1922a), Wheeler (1922b), Smith (1944), Weber (1952b), Wetterer (2012a)
Myrmicinae	*Cardiocondyla*	*Cardiocondylashuckardi* Forel, 1891		NK	Weber (1952b), Bolton (1982)
Myrmicinae	*Carebara*	*Carebaraacuta* (Weber, 1952)	Endemic	TO	Weber (1952b)
Myrmicinae	*Carebara*	*Carebaraamplacincta* Santschi, 1926	Endemic	HK	Santschi (1926a)
Myrmicinae	*Carebara*	*Carebaraamplaobscurithorax* Santschi, 1926	Endemic	HK	Santschi (1926a)
Myrmicinae	*Carebara*	*Carebaraamplarugosa* Santschi, 1928		HK	Santschi (1928b)
Myrmicinae	*Carebara*	*Carebaraampla* Santschi, 1912	Endemic	-	Santschi (1912), Wheeler (1922b)
Myrmicinae	*Carebara*	*Carebaraangolensis* (Santschi, 1914)		-	Braet and Taylor (2008)
Myrmicinae	*Carebara*	*Carebaraangolensiscongolensis* (Forel, 1916)	Endemic	-	Forel (1916), Wheeler (1922b)
Myrmicinae	*Carebara*	*Carebarafrontalis* (Weber, 1950)	Endemic	IT	Weber (1950)
Myrmicinae	*Carebara*	*Carebarajunodi* Forel, 1904		EQ, HK, HL, KC	Forel (1909), Forel (1913a), Wheeler (1922b), Santschi (1935a), Wheeler (1936)
Myrmicinae	*Carebara*	*Carebaralangi* Wheeler, 1922	Endemic	TO	Wheeler (1922a), Wheeler (1922b)
Myrmicinae	*Carebara*	*Carebaralatro* (Santschi, 1937)	Endemic	EQ	Santschi (1937a), Weber (1950)
Myrmicinae	*Carebara*	*Carebaramadibai* Fischer & Azorsa, 2014		-	Fischer et al. (2014)
Myrmicinae	*Carebara*	*Carebaraosborni* Wheeler, 1922	Endemic	HU	Wheeler (1922a), Wheeler (1922b), Wheeler (1936)
Myrmicinae	*Carebara*	*Carebarapetulca* (Wheeler, 1922)	Endemic	BC	Wheeler (1922a), Wheeler (1922b), Wheeler (1936)
Myrmicinae	*Carebara*	*Carebaratermitolestes* (Wheeler, 1918)		BC, TO	Wheeler (1922a), Wheeler (1922b), Wheeler (1936), Weber (1943), Bolton and Belshaw (1993), Van De Perre et al. (2018)
Myrmicinae	*Carebara*	*Carebaravidua* Smith, 1858		HK, HU, IT	Forel (1909), Forel (1913a), Wheeler (1922a), Wheeler (1922b)
Myrmicinae	*Cataulacus*	*Cataulacusbequaerti* Forel, 1913		EQ, HK, HL	Forel (1913a), Wheeler (1922b), Bolton (1974a), Bolton (1982)
Myrmicinae	*Cataulacus*	*Cataulacusbrevisetosus* Forel, 1901		BC, KN, SA	Bolton (1974a)
Myrmicinae	*Cataulacus*	*Cataulacuscestus* Bolton, 1982	Endemic	IT	Bolton (1982)
Myrmicinae	*Cataulacus*	*Cataulacusegenus* Santschi, 1911		HU, IT, KN, KS, MN, TO	Wheeler (1922a), Wheeler (1922b), Santschi (1924a), Santschi (1930a), Bolton (1974a), Bolton (1982)
Myrmicinae	*Cataulacus*	*Cataulacuselongatus* Santschi, 1924		EQ	Santschi (1924a)
Myrmicinae	*Cataulacus*	*Cataulacuserinaceus* Stitz, 1910		BU, EQ, HU, KN, KS, LO, MA, MN, MO, NK, SA, SU, TO, TU	Forel (1913a), Forel (1913c), Forel (1915), Forel (1916), Santschi (1917), Wheeler (1922a), Wheeler (1922b), Bolton (1974a), Bolton (1982), Van De Perre et al. (2018)
Myrmicinae	*Cataulacus*	*Cataulacusgreggi* Bolton, 1974		IT, TO	Bolton (1974a), Bolton (1982)
Myrmicinae	*Cataulacus*	*Cataulacusguineensis* Smith, 1853		BC, BU, EQ, HU, IT, KL, KN, KS, MN, MO, NK, TO	Forel (1916), Wheeler (1922a), Wheeler (1922b), Santschi (1924a), Bolton (1974a), Bolton (1982), Van De Perre et al. (2018)
Myrmicinae	*Cataulacus*	*Cataulacushuberi* André, 1890		EQ, HK, HU, IT, KS, MA, MN, MO, SU, TO	Santschi (1910), Forel (1913a), Wheeler (1922b), Santschi (1924a), Wheeler (1925), Bolton (1974a), Bolton (1982), Van De Perre et al. (2018)
Myrmicinae	*Cataulacus*	*Cataulacusinermis* Santschi, 1924	Endemic	KS	Santschi (1924a), Bolton (1974a), Bolton (1982)
Myrmicinae	*Cataulacus*	*Cataulacusjeanneli* Santschi, 1914		TO	Van De Perre et al. (2018)
Myrmicinae	*Cataulacus*	*Cataulacuskohli* Mayr, 1895		BC, EQ, HU, KS, MN, MO, TO	Santschi (1924a), Santschi (1930a), Bolton (1974a), Bolton (1982)
Myrmicinae	*Cataulacus*	*Cataulacuslobatus* Mayr, 1895		SA	Bolton (1974a), Bolton (1982)
Myrmicinae	*Cataulacus*	*Cataulacuslujae* Forel, 1911		HK, HL, HU, KS, SA, TO	Forel (1911a), Forel (1913a), Forel (1916), Wheeler (1922b), Bolton (1982)
Myrmicinae	*Cataulacus*	*Cataulacusmocquerysi* André, 1889		BC	Wheeler (1922b), Bolton (1974a), Bolton (1982)
Myrmicinae	*Cataulacus*	*Cataulacuspilosus* Santschi, 1920		TO	Santschi (1920b), Wheeler (1922b), Bolton (1974a), Bolton (1982)
Myrmicinae	*Cataulacus*	*Cataulacuspullus* Santschi, 1910		BC, EQ, TO	Santschi (1924a), Bolton (1974a), Bolton (1982), Van De Perre et al. (2018)
Myrmicinae	*Cataulacus*	*Cataulacuspygmaeus* André, 1890		BC, HU, IT, KN, KS, LU, MA, MN, MO, SA, SK, TO	Forel (1913c), Wheeler (1922a), Wheeler (1922b), Weber (1943), Bolton (1974a), Bolton (1982)
Myrmicinae	*Cataulacus*	*Cataulacusstriativentris* Santschi, 1924		HU, NU	Santschi (1924a), Bolton (1974a), Bolton (1982)
Myrmicinae	*Cataulacus*	*Cataulacustardus* Santschi, 1914		BC, KN, MN, TO	Santschi (1919b), Wheeler (1922b), Santschi (1924a), Bolton (1974a), Bolton (1982), Van De Perre et al. (2018)
Myrmicinae	*Cataulacus*	*Cataulacustheobromicola* Santschi, 1939	Endemic	HU	Bolton (1974a), Bolton (1982)
Myrmicinae	*Cataulacus*	*Cataulacustraegaordhi* Santschi, 1914		BC, IT, KN, MN, MO, TO	Santschi (1924a), Bernard (1953), Bolton (1982)
Myrmicinae	*Cataulacus*	*Cataulacusvorticus* Bolton, 1974		BC	Bolton (1974a)
Myrmicinae	*Cataulacus*	*Cataulacusweissi* Santschi, 1913		BC, KN, TO	Forel (1916), Wheeler (1922a), Wheeler (1922b), Santschi (1924a), Santschi (1935b), Bolton (1974a), Bolton (1982), Van De Perre et al. (2018)
Myrmicinae	*Crematogaster*	*Crematogasteracaciaegloriosa* Santschi, 1914	Endemic	BC	Santschi (1914), Wheeler (1922b), Soulie and Dicko (1965)
Myrmicinae	*Crematogaster*	*Crematogasteracaciaevictoriosa* Santschi, 1916		SU	Wheeler (1922a), Wheeler (1922b), Soulie and Dicko (1965)
Myrmicinae	*Crematogaster*	*Crematogasterafricanaalligatrix* Forel, 1911		BC, KS, SA, TO	Forel (1911b), Forel (1913c), Forel (1915), Wheeler (1922b), Soulie and Dicko (1965)
Myrmicinae	*Crematogaster*	*Crematogasterafricanacamena* Wheeler, 1922	Endemic	-	Forel (1916), Wheeler (1922b), Soulie and Dicko (1965)
Myrmicinae	*Crematogaster*	*Crematogasterafricana* Mayr, 1895		BC, KS, SA	Forel (1909), Forel (1913c), Wheeler (1922b), Santschi (1937a), Soulie and Dicko (1965)
Myrmicinae	*Crematogaster*	*Crematogasterafricanaschumanni* Mayr, 1895		KN	Wheeler (1922a), Wheeler (1922b), Soulie and Dicko (1965)
Myrmicinae	*Crematogaster*	*Crematogasterafricanastanleyi* Wheeler, 1922		IT, SA	Forel (1911b), Wheeler (1922b), Santschi (1930a), Soulie and Dicko (1965)
Myrmicinae	*Crematogaster*	*Crematogasterafricanastolonis* Santschi, 1937	Endemic	SA	Santschi (1937a)
Myrmicinae	*Crematogaster*	*Crematogasterafricanathoracica* Santschi, 1921	Endemic	SA	Santschi (1921)
Myrmicinae	*Crematogaster*	*Crematogasterafricanatibialis* Wheeler, 1922		NK	Wheeler (1922a), Wheeler (1922b), Soulie and Dicko (1965)
Myrmicinae	*Crematogaster*	*Crematogasterambigua* Santschi, 1926		EQ	Santschi (1926a)
Myrmicinae	*Crematogaster*	*Crematogasterangusticeps* Santschi, 1911		-	Santschi (1935a)
Myrmicinae	*Crematogaster*	*Crematogasterbequaertiatraplex* Wheeler, 1922	Endemic	HU	Wheeler (1922a), Wheeler (1922b), Soulie and Dicko (1965)
Myrmicinae	*Crematogaster*	*Crematogasterbequaerti* Forel, 1913		HK, HL	Forel (1913a), Wheeler (1922b), Weber (1964), Soulie and Dicko (1965)
Myrmicinae	*Crematogaster*	*Crematogasterbequaertigerardi* Santschi, 1915	Endemic	TA	Santschi (1915), Wheeler (1922b), Soulie and Dicko (1965)
Myrmicinae	*Crematogaster*	*Crematogasterbequaertiludia* Forel, 1913	Endemic	HK, HL	Forel (1913a), Wheeler (1922b), Soulie and Dicko (1965)
Myrmicinae	*Crematogaster*	*Crematogasterbuchnericomposita* Santschi, 1933	Endemic	SA	Santschi (1933)
Myrmicinae	*Crematogaster*	*Crematogasterbuchnerigraeteri* Forel, 1916		TO	Forel (1916), Wheeler (1922b), Soulie and Dicko (1965)
Myrmicinae	*Crematogaster*	*Crematogasterbuchneriuasina* Santschi, 1935		-	Santschi (1935b)
Myrmicinae	*Crematogaster*	*Crematogastercastaneaanalis* Santschi, 1910		BC, TO	Forel (1910b), Wheeler (1922a), Wheeler (1922b), Soulie and Dicko (1965)
Myrmicinae	*Crematogaster*	*Crematogastercastaneabusschodtsi* Emery, 1899		BC, KC, KN	Emery (1899), Wheeler (1922b), Santschi (1928a), Santschi (1930a), Santschi (1935a), Prins (1964), Soulie and Dicko (1965)
Myrmicinae	*Crematogaster*	*Crematogastercastaneainversa* Forel, 1907		SA	Forel (1911b), Wheeler (1922b), Soulie and Dicko (1965)
Myrmicinae	*Crematogaster*	*Crematogastercastaneamediorufa* Forel, 1907		-	Soulie and Dicko (1965)
Myrmicinae	*Crematogaster*	*Crematogastercastanearufonigra* Emery, 1895		HK	Forel (1913c), Wheeler (1922b), Prins (1963), Soulie and Dicko (1965)
Myrmicinae	*Crematogaster*	*Crematogastercastanea* Smith, 1858		SA	Wheeler (1922b), Soulie and Dicko (1965)
Myrmicinae	*Crematogaster*	*Crematogastercastaneayambatensis* Forel, 1913	Endemic	MO	Forel (1913c), Wheeler (1922b), Soulie and Dicko (1965)
Myrmicinae	*Crematogaster*	*Crematogasterclariventrisbiimpressa* Mayr, 1895		MN, NK, SA	Santschi (1921), Santschi (1933)
Myrmicinae	*Crematogaster*	*Crematogasterclariventris* Mayr, 1895		EQ, KS, SA, TO	Forel (1913c), Wheeler (1922b), Santschi (1930a), Santschi (1933), Santschi (1935a), Santschi (1935b), Soulie and Dicko (1965), Van De Perre et al. (2018)
Myrmicinae	*Crematogaster*	*Crematogastercoelestis* Santschi, 1911		KS, SA	Forel (1913a), Wheeler (1922b), Soulie and Dicko (1965)
Myrmicinae	*Crematogaster*	*Crematogasterconcava* Emery, 1899		BU, EQ, KG, KN, KS, TO	Emery (1899), Forel (1915), Forel (1916), Wheeler (1922a), Wheeler (1922b), Santschi (1935a), Soulie and Dicko (1965), Van De Perre et al. (2018)
Myrmicinae	*Crematogaster*	*Crematogasterdepressa* (Latreille, 1802)		BC, SA	Forel (1909), Wheeler (1922b), Santschi (1928a), Soulie and Dicko (1965)
Myrmicinae	*Crematogaster*	*Crematogasterdepressafuscipennis* Emery, 1899		BC, BU, EQ, HL, HU, KN, NU, TO	Emery (1899), Forel (1909), Santschi (1910), Forel (1910b), Forel (1913a), Forel (1913c), Wheeler (1922a), Wheeler (1922b), Santschi (1935a), Soulie and Dicko (1965)
Myrmicinae	*Crematogaster*	*Crematogasterexcisabomaella* Santschi, 1935	Endemic	BC	Santschi (1935a)
Myrmicinae	*Crematogaster*	*Crematogasterexcisa* Mayr, 1895		BC, HU, KL, MN, SU	Wheeler (1922a), Wheeler (1922b), Santschi (1935a), Soulie and Dicko (1965)
Myrmicinae	*Crematogaster*	*Crematogasterflaviventris* Santschi, 1910		HU, KL, SA	Santschi (1910), Santschi (1913a), Wheeler (1922a), Wheeler (1922b), Santschi (1935a), Weber (1943), Soulie and Dicko (1965)
Myrmicinae	*Crematogaster*	*Crematogasterforaminicepsstaitchi* Forel, 1915	Endemic	IT	Forel (1915), Wheeler (1922b), Soulie and Dicko (1965)
Myrmicinae	*Crematogaster*	*Crematogastergabonensis* Emery, 1899		BC, EQ, HL, MA	Santschi (1926a)
Myrmicinae	*Crematogaster*	*Crematogastergabonensisfuscitatis* Forel, 1913	Endemic	EQ, KN, KS, SA	Forel (1913c), Wheeler (1922b), Santschi (1937a), Soulie and Dicko (1965)
Myrmicinae	*Crematogaster*	*Crematogastergambiensis* André, 1889		IT	Forel (1913a), Wheeler (1922b), Soulie and Dicko (1965)
Myrmicinae	*Crematogaster*	*Crematogastergambiensissejuncta* Stitz, 1916	Endemic	HU	Stitz (1916), Wheeler (1922b), Santschi (1926a), Soulie and Dicko (1965)
Myrmicinae	*Crematogaster*	*Crematogastergerstaeckerikohliella* Santschi, 1918	Endemic	TO	Forel (1916), Wheeler (1922b), Soulie and Dicko (1965)
Myrmicinae	*Crematogaster*	*Crematogastergerstaeckerioraclum* Forel, 1913		HL, LU	Forel (1913a), Wheeler (1922b), Santschi (1937a), Soulie and Dicko (1965)
Myrmicinae	*Crematogaster*	*Crematogastergratiosa* Santschi, 1926		MA	Santschi (1926a), Santschi (1930a), Braet and Taylor (2008)
Myrmicinae	*Crematogaster*	*Crematogasterimpressa* Emery, 1899		BC, HU, KN, MA, NK, TO	Emery (1899), Forel (1909), Forel (1911b), Forel (1913c), Santschi (1913a), Forel (1916), Wheeler (1922a), Wheeler (1922b), Santschi (1935a), Soulie and Dicko (1965)
Myrmicinae	*Crematogaster*	*Crematogasterimpressamaynei* Forel, 1913	Endemic	KN	Forel (1911b), Forel (1913c), Wheeler (1922b), Soulie and Dicko (1965)
Myrmicinae	*Crematogaster*	*Crematogasterimpressasapora* Forel, 1916	Endemic	HU	Forel (1916), Wheeler (1922a), Wheeler (1922b), Soulie and Dicko (1965)
Myrmicinae	*Crematogaster*	*Crematogasterimpressicepsfrontalis* Wheeler, 1922	Endemic	BC	Wheeler (1922a), Wheeler (1922b), Soulie and Dicko (1965)
Myrmicinae	*Crematogaster*	*Crematogasterimpressicepslongiscapa* Stitz, 1916	Endemic	SU	Stitz (1916), Wheeler (1922b), Soulie and Dicko (1965)
Myrmicinae	*Crematogaster*	*Crematogasterimpressicepslujana* Forel, 1915	Endemic	SA	Forel (1915), Wheeler (1922b), Santschi (1930a), Soulie and Dicko (1965)
Myrmicinae	*Crematogaster*	*Crematogasterimpressiceps* Mayr, 1902		BC, HU, KS, TO	Wheeler (1922a), Wheeler (1922b), Santschi (1930a), Soulie and Dicko (1965)
Myrmicinae	*Crematogaster*	*Crematogasterjuventa* Santschi, 1926		BC, MN	Santschi (1926a), Santschi (1935a)
Myrmicinae	*Crematogaster*	*Crematogasterkasaiensis* Forel, 1913		KS, SA, TO	Forel (1913a), Forel (1916), Wheeler (1922b), Soulie and Dicko (1965), Braet and Taylor (2008)
Myrmicinae	*Crematogaster*	*Crematogasterkneri* Mayr, 1862		-	Wheeler (1922b), Prins (1963), Soulie and Dicko (1965)
Myrmicinae	*Crematogaster*	*Crematogasterkohli* Forel, 1909		IT, TO	Forel (1909), Forel (1915), Wheeler (1922b), Santschi (1937a), Soulie and Dicko (1965)
Myrmicinae	*Crematogaster*	*Crematogasterkohliwinkleri* Forel, 1909		EQ, HU, KS, MN, SA	Forel (1909), Forel (1911b), Forel (1913a), Forel (1913b), Forel (1916), Wheeler (1922b), Santschi (1937a), Soulie and Dicko (1965)
Myrmicinae	*Crematogaster*	*Crematogasterlaurenti* Forel, 1909		MN, SA, TO	Forel (1909), Wheeler (1922a), Wheeler (1922b), Santschi (1935a), Santschi (1937a), Soulie and Dicko (1965)
Myrmicinae	*Crematogaster*	*Crematogasterlaurentizeta* Emery, 1922	Endemic	KN, MA, SA, TO	Wheeler (1922a), Wheeler (1922b), Santschi (1937a), Soulie and Dicko (1965)
Myrmicinae	*Crematogaster*	*Crematogasterlibengensisrufula* Santschi, 1926		TO	Santschi (1926a)
Myrmicinae	*Crematogaster*	*Crematogasterlibengensis* Stitz, 1916		SU	Stitz (1916), Wheeler (1922b), Soulie and Dicko (1965), Braet and Taylor (2008)
Myrmicinae	*Crematogaster*	*Crematogasterlotti* Weber, 1943		KN	Weber (1964)
Myrmicinae	*Crematogaster*	*Crematogasterluctansliebknechti* Forel, 1915	Endemic	EQ, HU, TO	Forel (1915), Wheeler (1922a), Wheeler (1922b), Santschi (1933), Soulie and Dicko (1965)
Myrmicinae	*Crematogaster*	*Crematogastermargaritaebrevarmata* Forel, 1915	Endemic	MO, SA	Forel (1915), Wheeler (1922b), Santschi (1930a), Santschi (1935a), Soulie and Dicko (1965), Blaimer (2012)
Myrmicinae	*Crematogaster*	*Crematogastermargaritaecupida* Santschi, 1935	Endemic	KS	Santschi (1935a), Blaimer (2012)
Myrmicinae	*Crematogaster*	*Crematogastermargaritaelujae* Forel, 1913	Endemic	KS, SA	Forel (1913a), Wheeler (1922b), Soulie and Dicko (1965), Blaimer (2012)
Myrmicinae	*Crematogaster*	*Crematogastermenilekiiproserpina* Santschi, 1919	Endemic	BC	Wheeler (1922a), Wheeler (1922b), Soulie and Dicko (1965)
Myrmicinae	*Crematogaster*	*Crematogastermenilekiisatan* Forel, 1916	Endemic	-	Forel (1916), Wheeler (1922b), Soulie and Dicko (1965)
Myrmicinae	*Crematogaster*	*Crematogastermenilekiispuria* Forel, 1913		HK	Forel (1913a), Wheeler (1922b), Soulie and Dicko (1965)
Myrmicinae	*Crematogaster*	*Crematogastermuralti* Forel, 1910		KL, KS, MN, TO	Santschi (1930a), Santschi (1935a), Van De Perre et al. (2018)
Myrmicinae	*Crematogaster*	*Crematogasternigeriensiswilnigra* Forel, 1916	Endemic	TO	Forel (1916), Wheeler (1922b)
Myrmicinae	*Crematogaster*	*Crematogasternigrans* Forel, 1915		TO	Forel (1915), Wheeler (1922b), Soulie and Dicko (1965)
Myrmicinae	*Crematogaster*	*Crematogasteropacicepsclepens* Forel, 1913	Endemic	KN	Forel (1913c), Wheeler (1922b), Soulie and Dicko (1965)
Myrmicinae	*Crematogaster*	*Crematogasterpauciseta* Emery, 1899		TO	Forel (1915), Wheeler (1922b), Soulie and Dicko (1965), Braet and Taylor (2008), Van De Perre et al. (2018)
Myrmicinae	*Crematogaster*	*Crematogasterpaucisetagrossulior* Forel, 1916		TO	Forel (1916), Wheeler (1922b)
Myrmicinae	*Crematogaster*	*Crematogasterpetiolidens* Forel, 1916	Endemic	-	Forel (1916), Wheeler (1922b), Soulie and Dicko (1965)
Myrmicinae	*Crematogaster*	*Crematogasterpseudinermis* Viehmeyer, 1923		KN, KS, MN, MO	Santschi (1926a), Santschi (1935a)
Myrmicinae	*Crematogaster*	*Crematogasterrugosa* André, 1895		KS, TO	Santschi (1919c), Wheeler (1922b), Santschi (1935a), Soulie and Dicko (1965)
Myrmicinae	*Crematogaster*	*Crematogasterrugosior* Santschi, 1910		EQ, KS, TO	Forel (1916), Wheeler (1922a), Santschi (1926a), Soulie and Dicko (1965)
Myrmicinae	*Crematogaster*	*Crematogasterruspoliiatriscapis* Forel, 1915	Endemic	TO	Forel (1915), Forel (1916), Wheeler (1922b), Soulie and Dicko (1965)
Myrmicinae	*Crematogaster*	*Crematogastersantschii* Forel, 1913		KN, KS, SA, TO	Forel (1913a), Forel (1913c), Wheeler (1922b), Soulie and Dicko (1965), Blaimer (2012)
Myrmicinae	*Crematogaster*	*Crematogastersewelliiacis* Forel, 1913		KN	Forel (1913c), Wheeler (1922b), Soulie and Dicko (1965)
Myrmicinae	*Crematogaster*	*Crematogastersewelliimarnoi* Mayr, 1895		NU	Santschi (1910)
Myrmicinae	*Crematogaster*	*Crematogastersimilis* Stitz, 1911	Endemic	NK, TO	Stitz (1911), Wheeler (1922b), Soulie and Dicko (1965), Van De Perre et al. (2018)
Myrmicinae	*Crematogaster*	*Crematogastersolenopsidesflavida* Mayr, 1907		TO	Forel (1916)
Myrmicinae	*Crematogaster*	*Crematogasterstadelmannianguliceps* Stitz, 1916		BU	Santschi (1935a)
Myrmicinae	*Crematogaster*	*Crematogasterstadelmannidolichocephala* Santschi, 1911		HU, KL, MN, NU, SA, TO	Santschi (1910), Santschi (1911), Forel (1915), Wheeler (1922a), Wheeler (1922b), Soulie and Dicko (1965), Blaimer (2012)
Myrmicinae	*Crematogaster*	*Crematogasterstadelmanni* Mayr, 1895		KN, TO	Forel (1911b), Forel (1915), Forel (1916), Wheeler (1922a), Wheeler (1922b), Soulie and Dicko (1965)
Myrmicinae	*Crematogaster*	*Crematogasterstadelmanniovinodis* Stitz, 1916		SU	Stitz (1916), Wheeler (1922b), Soulie and Dicko (1965), Blaimer (2012)
Myrmicinae	*Crematogaster*	*Crematogasterstadelmannischereri* Forel, 1911		-	Blaimer (2012)
Myrmicinae	*Crematogaster*	*Crematogasterstadelmannispissata* Santschi, 1937	Endemic	SA	Santschi (1937a), Blaimer (2012)
Myrmicinae	*Crematogaster*	*Crematogasterstriatula* Emery, 1892		BC, NU	Santschi (1910), Wheeler (1922b), Santschi (1926a)
Myrmicinae	*Crematogaster*	*Crematogasterstriatulalangi* Santschi, 1926	Endemic	HU	Santschi (1926a)
Myrmicinae	*Crematogaster*	*Crematogasterstriatulaobstinata* Santschi, 1911		BC, BU, KN, MN, NU	Santschi (1911), Wheeler (1922a), Wheeler (1922b), Santschi (1926a), Soulie and Dicko (1965)
Myrmicinae	*Crematogaster*	*Crematogasterstriatulaomega* Santschi, 1935	Endemic	BC, EQ, KS, MN, MO	Santschi (1926a), Santschi (1935a)
Myrmicinae	*Crematogaster*	*Crematogastertheta* Forel, 1911	Endemic	BU, EQ, HU, KS, SA, TO	Forel (1911b), Forel (1913a), Forel (1913b), Forel (1913c), Forel (1916), Wheeler (1922a), Wheeler (1922b), Santschi (1933), Santschi (1935a), Santschi (1937a), Soulie and Dicko (1965)
Myrmicinae	*Crematogaster*	*Crematogastertransiens* Forel, 1913	Endemic	HL, TA, TO	Forel (1913a), Santschi (1915), Wheeler (1922a), Wheeler (1922b), Soulie and Dicko (1965), Van De Perre et al. (2018)
Myrmicinae	*Crematogaster*	*Crematogastertricolor* Gerstäcker, 1859		SA	Forel (1911b)
Myrmicinae	*Crematogaster*	*Crematogasterwasmanni* Santschi, 1910		SA	Santschi (1910), Wheeler (1922b), Soulie and Dicko (1965), Braet and Taylor (2008)
Myrmicinae	*Crematogaster*	*Crematogasterwellmaniluciae* Forel, 1913		IT	Forel (1913c), Forel (1916), Wheeler (1922b), Soulie and Dicko (1965)
Myrmicinae	*Crematogaster*	*Crematogasterwellmaniweissi* Santschi, 1910		NU	Santschi (1910), Wheeler (1922b)
Myrmicinae	*Crematogaster*	*Crematogasterwilwerthiconfusa* Santschi, 1911		NU	Santschi (1911), Wheeler (1922b)
Myrmicinae	*Crematogaster*	*Crematogasterwilwerthifauconneti* Forel, 1910	Endemic	MA, TO	Forel (1910b), Wheeler (1922b), Soulie and Dicko (1965)
Myrmicinae	*Crematogaster*	*Crematogasterwilwerthi* Santschi, 1910	Endemic	BC	Santschi (1910), Wheeler (1922b), Soulie and Dicko (1965)
Myrmicinae	*Cyphoidris*	*Cyphoidrisspinosa* Weber, 1952		NK	Weber (1952b), Bolton (1981a), Taylor (2009)
Myrmicinae	*Dicroaspis*	*Dicroaspiscryptocera* Emery, 1908		TO	Emery (1908), Wheeler (1922b), Bolton (1981b)
Myrmicinae	*Dicroaspis*	*Dicroaspislaevidens* (Santschi, 1919)		TO	Santschi (1919c), Wheeler (1922b), Bolton (1981b), Collingwood and Van Harten (2005)
Myrmicinae	*Melissotarsus*	*Melissotarsusemeryi* Forel, 1907		KG	Bolton (1982)
Myrmicinae	*Melissotarsus*	*Melissotarsusweissi* Santschi, 1910		KS, LO	Santschi (1919c), Wheeler (1922b), Santschi (1923), Bolton (1982)
Myrmicinae	*Meranoplus*	*Meranoplusclypeatus* Bernard, 1953		HU	Bolton (1981b)
Myrmicinae	*Meranoplus*	*Meranoplusinermis* Emery, 1895		HU, LU, TO	Wheeler (1922a), Wheeler (1922b), Weber (1943), Bolton (1981b)
Myrmicinae	*Meranoplus*	*Meranoplusmagrettii* André, 1884		TA	Santschi (1915), Wheeler (1922b), Bolton (1981b)
Myrmicinae	*Meranoplus*	*Meranoplusnanus* André, 1892		HU, LU	Bolton (1981b)
Myrmicinae	*Microdaceton*	*Microdacetontibialis* Weber, 1952		IT, TO	Weber (1952b), Bolton (1983), Bolton (2000)
Myrmicinae	*Monomorium*	*Monomoriumaffabile* Santschi, 1926	Endemic	NU	Santschi (1926a), Bolton (1987)
Myrmicinae	*Monomorium*	*Monomoriumafrum* André, 1884		BU, HL, HU	Forel (1913a), Wheeler (1922a), Wheeler (1922b), Bolton (1987)
Myrmicinae	*Monomorium*	*Monomoriumaltinode* Santschi, 1910		BC, TA	Santschi (1920a), Wheeler (1922b), Santschi (1935a), Bolton (1987), Braet and Taylor (2008)
Myrmicinae	*Monomorium*	*Monomoriumangustinode* Forel, 1913		HK	Forel (1913a), Wheeler (1922b), Bolton (1987), Braet and Taylor (2008)
Myrmicinae	*Monomorium*	*Monomoriumbequaerti* Forel, 1913		HK, KS	Forel (1913a), Wheeler (1922b), Santschi (1926a), Bolton (1987)Bolton 1987
Myrmicinae	*Monomorium*	*Monomoriumbicolor* Emery, 1877		HU, IT, KN, KS, TO	Wheeler (1922a), Wheeler (1922b), Santschi (1926a), Bolton (1987)
Myrmicinae	*Monomorium*	*Monomoriumcaptator* Santschi, 1936		-	Bolton (1987), Braet and Taylor (2008)
Myrmicinae	*Monomorium*	*Monomoriumegens* Forel, 1910		IT, KS, TA	Santschi (1926a), Bolton (1987)
Myrmicinae	*Monomorium*	*Monomoriumexiguum* Forel, 1894		KN, TO	Forel (1913c), Forel (1916), Wheeler (1922b), Finzi (1939), Bolton (1987), Heterick (2006), Sharaf et al. (2018)
Myrmicinae	*Monomorium*	*Monomoriumfloricola* (Jerdon, 1851)	Exotic	-	Wetterer (2010)
Myrmicinae	*Monomorium*	*Monomoriumgabrielense* Forel, 1916		TO	Forel (1916), Wheeler (1922b), Bolton (1987)
Myrmicinae	*Monomorium*	*Monomoriuminquietum* Santschi, 1926		HU	Santschi (1926a), Bolton (1987)
Myrmicinae	*Monomorium*	*Monomoriummadecassum* Forel, 1892		TO	Forel (1905), Wheeler (1922b), Santschi (1928b), Bolton (1987), Heterick (2006)
Myrmicinae	*Monomorium*	*Monomoriummalatu* Bolton, 1987		BC, HU	Bolton (1987)
Myrmicinae	*Monomorium*	*Monomoriumopacum* Forel, 1913		HK	Forel (1913a), Wheeler (1922b), Bolton (1987)
Myrmicinae	*Monomorium*	*Monomoriumpharaonis* (Linnaeus, 1758)		BC, SA, TO	Forel (1913c), Wheeler (1922a), Wheeler (1922b)
Myrmicinae	*Monomorium*	*Monomoriumrosae* Santschi, 1920		BC, SK	Santschi (1920a), Wheeler (1922b), Bolton (1987)
Myrmicinae	*Monomorium*	*Monomoriumstrangulatum* Santschi, 1921		IT	Bolton (1987)
Myrmicinae	*Monomorium*	*Monomoriumsubdentatum* Forel, 1913	Endemic	HK	Forel (1913a), Wheeler (1922b), Bolton (1987)
Myrmicinae	*Monomorium*	*Monomoriumvaguum* Santschi, 1930		KN	Santschi (1930a), Bolton (1987)
Myrmicinae	*Myrmicaria*	*Myrmicariaexigua* André, 1890		MO	Forel (1916), Wheeler (1922b), Santschi (1935a)
Myrmicinae	*Myrmicaria*	*Myrmicariaexiguakisangani* Wheeler, 1922		TO	Wheeler (1922a), Wheeler (1922b), Santschi (1925)
Myrmicinae	*Myrmicaria*	*Myrmicariaexiguaobscura* Santschi, 1920		BC	Santschi (1920b), Wheeler (1922b), Santschi (1925)
Myrmicinae	*Myrmicaria*	*Myrmicariaexiguapulla* Santschi, 1920	Endemic	MA, TO	Santschi (1920b), Wheeler (1922b), Santschi (1925)
Myrmicinae	*Myrmicaria*	*Myrmicariaexiguarufiventris* Forel, 1915	Endemic	IT, KL, TO	Forel (1915), Forel (1916), Wheeler (1922b), Santschi (1925)
Myrmicinae	*Myrmicaria*	*Myrmicariafumatalinearis* Santschi, 1925	Endemic	MN	Santschi (1925)
Myrmicinae	*Myrmicaria*	*Myrmicariafumata* Santschi, 1916		SU	Bernard (1953)
Myrmicinae	*Myrmicaria*	*Myrmicariafusca* Stitz, 1911		IT, NK	Stitz (1911), Wheeler (1922b)
Myrmicinae	*Myrmicaria*	*Myrmicariairregularis* Santschi, 1925		BC, EQ, KS, SA	Santschi (1925)
Myrmicinae	*Myrmicaria*	*Myrmicarianatalensis* (Smith, 1858)		LO, SA, TA	Santschi (1925), Santschi (1930a), Santschi (1933)
Myrmicinae	*Myrmicaria*	*Myrmicarianatalensiseumenoides* (Gerstäcker, 1859)		IT, KN, NK, SK, TO	Stitz (1911), Forel (1913c), Forel (1916), Wheeler (1922b), Prins (1963)
Myrmicinae	*Myrmicaria*	*Myrmicarianatalensistaeniata* Santschi, 1930		HK	Santschi (1930b)
Myrmicinae	*Myrmicaria*	*Myrmicariaopaciventriscongolensis* Forel, 1909		BC, HK, IT, KN, MN, MO, NK, SA	Forel (1909), Forel (1911b), Forel (1913a), Forel (1913c), Wheeler (1922a), Wheeler (1922b), Santschi (1925), Wheeler (1925), Weber (1964)
Myrmicinae	*Myrmicaria*	*Myrmicariaopaciventriscrucheti* Santschi, 1925		HU, KC, KN, TO	Wheeler (1922a), Wheeler (1922b), Santschi (1925)
Myrmicinae	*Myrmicaria*	*Myrmicariaopaciventris* Emery, 1893		BC, BU, HU, KN, MA, NK, SU, TO	Santschi (1910), Stitz (1910), Forel (1911b), Forel (1913c), Stitz (1916), Wheeler (1922a), Wheeler (1922b)
Myrmicinae	*Myrmicaria*	*Myrmicariaopaciventrismesonotalis* Santschi, 1925	Endemic	HU, SA, SU	Santschi (1925)
Myrmicinae	*Myrmicaria*	*Myrmicariasalambo* Wheeler, 1922		HU	Wheeler (1922a), Wheeler (1922b)
Myrmicinae	*Myrmicaria*	*Myrmicariastriatabuttgenbachi* Forel, 1913	Endemic	HK	Forel (1913a), Wheeler (1922b), Santschi (1925)
Myrmicinae	*Myrmicaria*	*Myrmicariastriata* Stitz, 1911		HK	Forel (1913a), Wheeler (1922b)
Myrmicinae	*Myrmicaria*	*Myrmicariastriatula* Santschi, 1925		BC	Santschi (1935a)
Myrmicinae	*Nesomyrmex*	*Nesomyrmexevelynae* (Forel, 1916)		TO	Forel (1916), Wheeler (1922b), Bolton (1982), Hita Garcia et al. (2017a)
Myrmicinae	*Nesomyrmex*	*Nesomyrmexgrisoni* (Forel, 1916)		TO	Forel (1916), Wheeler (1922b), Bolton (1982), Hita Garcia et al. (2017a)
Myrmicinae	*Nesomyrmex*	*Nesomyrmexinnocens* (Forel, 1913)		HK	Forel (1913a), Wheeler (1922b), Bolton (1982), Hita Garcia et al. (2017a)
Myrmicinae	*Pheidole*	*Pheidolealbidula* Santschi, 1928		KG	Santschi (1928b), Braet and Taylor (2008)
Myrmicinae	*Pheidole*	*Pheidoleaurivilliiattenuata* Santschi, 1910		HU, MA, NK	Wheeler (1922a), Wheeler (1922b)
Myrmicinae	*Pheidole*	*Pheidoleaurivilliikasaiensis* Forel, 1911		SA, TO	Forel (1911b), Forel (1916), Wheeler (1922b)
Myrmicinae	*Pheidole*	*Pheidoleaurivillii* Mayr, 1896		MN	Santschi (1935a)
Myrmicinae	*Pheidole*	*Pheidoleaurivilliirubricalva* Forel, 1915	Endemic	TO	Forel (1915), Wheeler (1922b)
Myrmicinae	*Pheidole*	*Pheidolebatrachorum* Wheeler, 1922		BU	Wheeler (1922a), Wheeler (1922b), Fischer et al. (2012)
Myrmicinae	*Pheidole*	*Pheidolebequaerti* Forel, 1913		HL	Forel (1913a), Wheeler (1922b)
Myrmicinae	*Pheidole*	*Pheidolebuchholzi* Mayr, 1901		KL, MO	Forel (1916), Wheeler (1922b), Santschi (1935a)
Myrmicinae	*Pheidole*	*Pheidolecaffrabayeri* Forel, 1916		NK	Forel (1916), Wheeler (1922b), Braet and Taylor (2008)
Myrmicinae	*Pheidole*	*Pheidolecaffrasenilifrons* Wheeler, 1922		HU	Wheeler (1922a), Wheeler (1922b), Braet and Taylor (2008)
Myrmicinae	*Pheidole*	*Pheidolechristinae* Fischer, Hita Garcia & Peters, 2012		IT	Fischer et al. (2012)
Myrmicinae	*Pheidole*	*Pheidolecorticicola* Santschi, 1910		NU	Santschi (1910), Wheeler (1922b)
Myrmicinae	*Pheidole*	*Pheidolecrassinodaruspolii* Emery, 1897		IT	Wheeler (1922b)
Myrmicinae	*Pheidole*	*Pheidoledea* Santschi, 1921		NK	Fischer et al. (2012), Gómez et al. (2022)
Myrmicinae	*Pheidole*	*Pheidoleexcellensfulvobasalis* Santschi, 1921		BU	Santschi (1921)
Myrmicinae	*Pheidole*	*Pheidoleexcellensweissi* Santschi, 1910		BC	Santschi (1930a)
Myrmicinae	*Pheidole*	*Pheidoleglabrella* Fischer, Hita Garcia & Peters, 2012		BU	Fischer et al. (2012), Gómez et al. (2022)
Myrmicinae	*Pheidole*	*Pheidolekohli* Mayr, 1901		HU, TO	Wheeler (1922a), Wheeler (1922b)
Myrmicinae	*Pheidole*	*Pheidoleliengmei* Forel, 1894		-	Weber (1943)
Myrmicinae	*Pheidole*	*Pheidoleliengmeishinsendensis* Forel, 1913		HK	Forel (1913a), Wheeler (1922b)
Myrmicinae	*Pheidole*	*Pheidolemegacephala* (Fabricius, 1793)		BC, BU, HU, SU, TO	Wheeler (1922a), Wheeler (1922b), Wetterer (2012c)
Myrmicinae	*Pheidole*	*Pheidolemegacephalaatrox* Forel, 1913		HK, HL	Forel (1913a), Santschi (1937a)
Myrmicinae	*Pheidole*	*Pheidolemegacephalailgii* Forel, 1907		IT	Wheeler (1922a), Wheeler (1922b)
Myrmicinae	*Pheidole*	*Pheidolemegacephalaimpressifrons* Wasmann, 1905		HK	Forel (1913a), Forel (1916), Wheeler (1922b), Prins (1963)
Myrmicinae	*Pheidole*	*Pheidolemegacephalamelancholica* Santschi, 1912		EQ, HU, TO	Wheeler (1922a), Wheeler (1922b), Santschi (1937a)
Myrmicinae	*Pheidole*	*Pheidolemegacephalankomoana* Forel, 1916		TO	Forel (1916), Wheeler (1922b)
Myrmicinae	*Pheidole*	*Pheidoleminimamalelana* Wheeler, 1922	Endemic	BC	Wheeler (1922a), Wheeler (1922b)
Myrmicinae	*Pheidole*	*Pheidoleminimamylognatha* Wheeler, 1922		BC	Wheeler (1922a), Wheeler (1922b), Braet and Taylor (2008)
Myrmicinae	*Pheidole*	*Pheidoleneokohli* Wilson, 1984	Endemic	TO	Wilson (1984), Lampe et al. (2006)
Myrmicinae	*Pheidole*	*Pheidolephilippi* Emery, 1915		TO	Van De Perre et al. (2018)
Myrmicinae	*Pheidole*	*Pheidolepulchella* Santschi, 1910		BU	Wheeler (1922a), Wheeler (1922b), Fischer et al. (2012)
Myrmicinae	*Pheidole*	*Pheidolepunctulata* Mayr, 1866		BC, BU, HK, HL, HU, KC, KN, KS, LU, MN, SA, SK, SU, TO	Forel (1909), Forel (1913a), Forel (1913c), Wheeler (1922a), Wheeler (1922b), Santschi (1935a), Prins (1963), Weber (1964)
Myrmicinae	*Pheidole*	*Pheidolepunctulatasubatrox* Santschi, 1937	Endemic	EQ, TO	Santschi (1937a)
Myrmicinae	*Pheidole*	*Pheidoleretronitens* Santschi, 1930		KS	Santschi (1930a)
Myrmicinae	*Pheidole*	*Pheidolesaxicola* Wheeler, 1922		BC, SU	Wheeler (1922a), Wheeler (1922b), Braet and Taylor (2008)
Myrmicinae	*Pheidole*	*Pheidoleschoutedeni* Forel, 1913		HK	Forel (1913a), Wheeler (1922b), Braet and Taylor (2008)
Myrmicinae	*Pheidole*	*Pheidoleschoutedeniplatycephala* Stitz, 1916	Endemic	SU	Stitz (1916), Wheeler (1922b)
Myrmicinae	*Pheidole*	*Pheidolesculpturataberthoudi* Forel, 1894		BC, KN, NU	Santschi (1910), Wheeler (1922b)
Myrmicinae	*Pheidole*	*Pheidolesculpturatadignata* Santschi, 1915		BC, NU	Santschi (1915), Wheeler (1922b)
Myrmicinae	*Pheidole*	*Pheidolesculpturata* Mayr, 1866		BC, KN, NU	Wheeler (1922b)
Myrmicinae	*Pheidole*	*Pheidolesculpturatawelgelegenensis* Forel, 1913		HK	Forel (1913a), Forel (1913c), Wheeler (1922b)
Myrmicinae	*Pheidole*	*Pheidolesetosa* Fischer, Hita Garcia & Peters, 2012	Endemic	IT	Fischer et al. (2012)
Myrmicinae	*Pheidole*	*Pheidolespeculiferabispecula* Santschi, 1930	Endemic	HU	Santschi (1930a)
Myrmicinae	*Pheidole*	*Pheidolespeculiferacubangensis* Forel, 1901		MO	Forel (1916), Wheeler (1922b)
Myrmicinae	*Pheidole*	*Pheidolespeculifera* Emery, 1877		HU, IT, TO	Wheeler (1922a), Wheeler (1922b), Weber (1964), Van De Perre et al. (2018)
Myrmicinae	*Pheidole*	*Pheidoletenuinodis* Mayr, 1901		TO	Van De Perre et al. (2018)
Myrmicinae	*Pheidole*	*Pheidolevanderveldi* Forel, 1913		HL	Forel (1913a), Arnold (1920), Wheeler (1922b)
Myrmicinae	*Pristomyrmex*	*Pristomyrmexafricanus* Karavaiev, 1931		BC, IT, KN, NK, TO	Weber (1952b), Bolton (1981a), Wang (2003), Laciny et al. (2016)
Myrmicinae	*Pristomyrmex*	*Pristomyrmexorbiceps* (Santschi, 1914)		BU, MN	Santschi (1924a), Weber (1952b), Bolton (1981a), Wang (2003)
Myrmicinae	*Pristomyrmex*	*Pristomyrmextrogor* Bolton, 1981		SK	Bolton (1981a), Wang (2003)
Myrmicinae	*Solenopsis*	*Solenopsisgeminata* (Fabricius, 1804)	Exotic	MA, NU	Santschi (1915), Wheeler (1922b)
Myrmicinae	*Solenopsis*	*Solenopsispunctaticepscaffra* Forel, 1894		HK	Forel (1913a), Wheeler (1922b)
Myrmicinae	*Solenopsis*	*Solenopsispunctaticepskibaliensis* Wheeler, 1922	Endemic	HK, HU	Wheeler (1922a), Wheeler (1922b), Weber (1964)
Myrmicinae	*Solenopsis*	*Solenopsispunctaticeps* Mayr, 1865		KN, MA	Forel (1911b), Wheeler (1922b), Santschi (1935a)
Myrmicinae	*Solenopsis*	*Solenopsisugandensiscongolensis* Santschi, 1935	Endemic	KS	Santschi (1935a)
Myrmicinae	*Strumigenys*	*Strumigenysbehasyla* (Bolton, 1983)		IT	Bolton (2000)
Myrmicinae	*Strumigenys*	*Strumigenysbellatrix* (Bolton, 2000)		IT	Bolton (2000)
Myrmicinae	*Strumigenys*	*Strumigenysbequaerti* Santschi, 1923		NK	Santschi (1923), Brown (1952), Bolton (2000)
Myrmicinae	*Strumigenys*	*Strumigenysbernardi* Brown, 1960		IT, TO	Brown (1960), Bolton (1983), Bolton (2000)
Myrmicinae	*Strumigenys*	*Strumigenysbitheria* Bolton, 1983		TO	Bolton (2000)
Myrmicinae	*Strumigenys*	*Strumigenyscavinasis* (Brown, 1950)		IT, NK	Brown (1950b), Weber (1952a), Brown (1953), Bolton (1983), Bolton (2000)
Myrmicinae	*Strumigenys*	*Strumigenysconcolor* Santschi, 1914		HU, IT, NK	Bolton (1983), Bolton (2000)
Myrmicinae	*Strumigenys*	*Strumigenysdextra* Brown, 1954		IT	Bolton (2000)
Myrmicinae	*Strumigenys*	*Strumigenysdotaja* (Bolton, 1983)		IT	Bolton (2000)
Myrmicinae	*Strumigenys*	*Strumigenysenkara* (Bolton, 1983)		IT	Bolton (2000)
Myrmicinae	*Strumigenys*	*Strumigenysfenkara* (Bolton, 1983)		KN	Bolton (2000)
Myrmicinae	*Strumigenys*	*Strumigenyshensekta* (Bolton, 1983)		IT	Bolton (2000)
Myrmicinae	*Strumigenys*	*Strumigenysinquilina* (Bolton, 1983)	Endemic	SK	Bolton (1983), Bolton (2000)
Myrmicinae	*Strumigenys*	*Strumigenysludovici* Forel, 1904		IT, NK, TO	Brown (1952), Weber (1952a), Bolton (1983), Bolton (2000)
Myrmicinae	*Strumigenys*	*Strumigenyslujae* Forel, 1902		BC, HU, IT, MA, MO, NK, SK, TA, TO	Santschi (1919c), Wheeler (1922b), Santschi (1923), Brown (1952), Weber (1952a), Bernard (1953), Bolton (1983), Bolton (2000)
Myrmicinae	*Strumigenys*	*Strumigenysmalaplax* (Bolton, 1983)		TO	Bolton (1983)
Myrmicinae	*Strumigenys*	*Strumigenysmaynei* Forel, 1916		EQ, MO, NK, TO	Brown (1952)
Myrmicinae	*Strumigenys*	*Strumigenysmormo* (Bolton, 2000)	Endemic	-	Bolton (2000)
Myrmicinae	*Strumigenys*	*Strumigenysninda* (Bolton, 1983)		IT	Bolton (2000)
Myrmicinae	*Strumigenys*	*Strumigenyspetiolata* Bernard, 1953		IT, NK	Bolton (2000)
Myrmicinae	*Strumigenys*	*Strumigenyspiliversa* (Bolton, 2000)	Endemic	IT	Bolton (2000)
Myrmicinae	*Strumigenys*	*Strumigenysrelahyla* Bolton, 1983		IT	Bolton (1983), Bolton (2000)
Myrmicinae	*Strumigenys*	*Strumigenysrogeri* Emery, 1890		KL	Bolton (2000), Wetterer (2012b)
Myrmicinae	*Strumigenys*	*Strumigenysroomi* (Bolton, 1972)		IT	Bolton (2000)
Myrmicinae	*Strumigenys*	*Strumigenysrufobrunea* Santschi, 1914		IT	Brown (1954)
Myrmicinae	*Strumigenys*	*Strumigenyssarissa* Bolton, 1983		SK	Bolton (1983)
Myrmicinae	*Strumigenys*	*Strumigenysserrula* Santschi, 1910		HU, IT, KS, NK, TO	Santschi (1923), Brown (1952), Weber (1952a), Bolton (1983), Bolton (2000)
Myrmicinae	*Strumigenys*	*Strumigenyssimoni* Emery, 1895		HK, TO	Forel (1913a), Forel (1916), Wheeler (1922b), Brown (1952), Bolton (1983), Bolton (2000)
Myrmicinae	*Strumigenys*	*Strumigenyssistrura* (Bolton, 1983)		IT	Bolton (2000)
Myrmicinae	*Strumigenys*	*Strumigenystacta* (Bolton, 1983)		IT, TO	Bolton (1983), Bolton (2000)
Myrmicinae	*Strumigenys*	*Strumigenystethepa* (Bolton, 2000)		IT	Bolton (2000)
Myrmicinae	*Strumigenys*	*Strumigenysweberi* (Brown, 1959)		HU	Brown (1959), Bolton (1983), Bolton (2000)
Myrmicinae	*Strumigenys*	*Strumigenysxenohyla* Bolton, 1983		IT	Bolton (1983)
Myrmicinae	*Syllophopsis*	*Syllophopsiscryptobia* Santschi, 1921		-	Bolton (1987), Heterick (2006), Sharaf and Aldawood (2013)
Myrmicinae	*Tetramorium*	*Tetramoriumaculeatum* (Mayr, 1866)		BC, BU, EQ, HU, IT, KN, KS, MO, NK, SA, SU, TO	Forel (1901a), Emery (1908), Forel (1909), Forel (1913c), Forel (1915), Forel (1916), Stitz (1916), Santschi (1919b), Wheeler (1922a), Wheeler (1922b), Santschi (1924a), Santschi (1933), Santschi (1935a), Bolton (1980)
Myrmicinae	*Tetramorium*	*Tetramoriumafricanum* (Mayr, 1866)		BU, KN, TO	Forel (1911b), Forel (1916), Bolton (1980)
Myrmicinae	*Tetramorium*	*Tetramoriumagna* (Santschi, 1935)	Endemic	EQ	Santschi (1935a), Bolton (1976)
Myrmicinae	*Tetramorium*	*Tetramoriumakengense* (Wheeler, 1922)		BU, KS	Wheeler (1922a), Wheeler (1922b), Santschi (1935a)
Myrmicinae	*Tetramorium*	*Tetramoriumamissum* Bolton, 1980	Endemic	SK	Bolton (1980)
Myrmicinae	*Tetramorium*	*Tetramoriumangulinode* Santschi, 1910		EQ, HU, IT, KG, KN, NK, TO	Forel (1911b), Forel (1913c), Wheeler (1922a), Wheeler (1922b), Santschi (1928b), Bolton (1980)
Myrmicinae	*Tetramorium*	*Tetramoriumbequaerti* Forel, 1913		HK, HL	Forel (1913a), Arnold (1917), Wheeler (1922b), Bolton (1980)
Myrmicinae	*Tetramorium*	*Tetramoriumboltoni* Hita Garcia, Fischer & Peters, 2010		IT, KL, TO
					Hita Garcia et al. (2010), Hita Garcia and Fischer (2014)
Myrmicinae	*Tetramorium*	*Tetramoriumbrevispinosum* (Stitz, 1910)		IT, MO, NK, TO	Santschi (1935a), Bolton (1976)
Myrmicinae	*Tetramorium*	*Tetramoriumbuthrum* Bolton, 1980		HU	Bolton (1980)
Myrmicinae	*Tetramorium*	*Tetramoriumcaldarium* (Roger, 1857)		HU	Wheeler (1922a), Wheeler (1922b), Bolton (1979), Bolton (1980), Wetterer and Hita Garcia (2015)
Myrmicinae	*Tetramorium*	*Tetramoriumcandidum* Bolton, 1980		SK	Bolton (1980)
Myrmicinae	*Tetramorium*	*Tetramoriumcapillosum* Bolton, 1980		IT	Hita Garcia and Fisher (2013)
Myrmicinae	*Tetramorium*	*Tetramoriumcoloreum* Mayr, 1901		IT	Brown (1957a), Bolton (1980)
Myrmicinae	*Tetramorium*	*Tetramoriumcristatum* Stitz, 1910		BC, HU, SA	Wheeler (1922a), Wheeler (1922b), Santschi (1924a), Weber (1943), Bolton (1980)
Myrmicinae	*Tetramorium*	*Tetramoriumdolichosum* Bolton, 1980	Endemic	LU	Bolton (1980)
Myrmicinae	*Tetramorium*	*Tetramoriumdumezi* Menozzi, 1942		BC, TO	Wheeler (1922a), Wheeler (1922b), Bolton (1980)
Myrmicinae	*Tetramorium*	*Tetramoriumedouardi* Forel, 1894		SK, TO	Stitz (1911), Wheeler (1922b), Santschi (1928b), Levieux (1972), Bolton (1980), Hita Garcia et al. (2010)
Myrmicinae	*Tetramorium*	*Tetramoriumgabonense* (André, 1892)		BC, BU, HU, IT, MO, NK, SA, SU, TO	Menozzi (1932)
Myrmicinae	*Tetramorium*	*Tetramoriumgazense* Arnold, 1958		HK, LU	Bolton (1980)
Myrmicinae	*Tetramorium*	*Tetramoriumgegaimi* Forel, 1916		TO	Forel (1916), Wheeler (1922b), Bolton (1980)
Myrmicinae	*Tetramorium*	*Tetramoriumglobulinode* (Mayr, 1901)		-	Forel (1916), Wheeler (1922b), Bolton (1976), Bolton (1986)
Myrmicinae	*Tetramorium*	*Tetramoriumguineense* (Bernard, 1953)		HK, HU, IT	Forel (1913a), Wheeler (1922a), Wheeler (1922b), Bolton (1980), Hita Garcia et al. (2010), Hita Garcia and Fischer (2014)
Myrmicinae	*Tetramorium*	*Tetramoriumhumbloti* Forel, 1891		HK	Santschi (1935a), Bolton (1980)
Myrmicinae	*Tetramorium*	*Tetramoriuminezulae* (Forel, 1914)		LU	Bolton (1976)
Myrmicinae	*Tetramorium*	*Tetramoriumkestrum* Bolton, 1980		HU	Bolton (1980)
Myrmicinae	*Tetramorium*	*Tetramoriumkhyarum* Bolton, 1980		BC, SU	Bolton (1980)
Myrmicinae	*Tetramorium*	*Tetramoriumlucayanum* Wheeler, 1905		KN	Forel (1909), Wheeler (1922b), Brown (1964a), Bolton (1979), Bolton (1980), Wetterer (2013b)
Myrmicinae	*Tetramorium*	*Tetramoriummeressei* Forel, 1916		NK	Forel (1916), Wheeler (1922a), Wheeler (1922b), Bolton (1980)
Myrmicinae	*Tetramorium*	*Tetramoriummuralti* Forel, 1910		BU, IT, LO	Santschi (1919c), Wheeler (1922b), Hita Garcia et al. (2010)
Myrmicinae	*Tetramorium*	*Tetramoriummuscorum* Arnold, 1926		IT, NK	Bolton (1976)
Myrmicinae	*Tetramorium*	*Tetramoriumnodiferum* (Emery, 1901)		HU	Santschi (1924a)
Myrmicinae	*Tetramorium*	*Tetramoriumnotiale* Bolton, 1980		LU	Bolton (1980)
Myrmicinae	*Tetramorium*	*Tetramoriumoccidentale* (Santschi, 1916)		BU, IT, KS, LO	Bolton (1980)
Myrmicinae	*Tetramorium*	*Tetramoriumopacum* (Emery, 1909)		BC, NK	Forel (1909), Forel (1916), Santschi (1916), Wheeler (1922a), Wheeler (1922b), Brown (1964b), Bolton (1976), Bolton (1986)
Myrmicinae	*Tetramorium*	*Tetramoriumpeutli* Forel, 1916		IT, MN, TO	Forel (1916), Wheeler (1922b), Santschi (1924a), Bolton (1980)
Myrmicinae	*Tetramorium*	*Tetramoriumphasias* Forel, 1914		KN	Bolton (1980)
Myrmicinae	*Tetramorium*	*Tetramoriumpostpetiolatum* Santschi, 1919	Endemic	IT, LO	Santschi (1919c), Wheeler (1922b), Brown (1957a), Bolton (1980)
Myrmicinae	*Tetramorium*	*Tetramoriumpulcherrimum* (Donisthorpe, 1945)		IT	Bolton (1976)
Myrmicinae	*Tetramorium*	*Tetramoriumpullulum* Santschi, 1924		HU, NK	Santschi (1924a), Santschi (1935a), Bolton (1980)
Myrmicinae	*Tetramorium*	*Tetramoriumquadridentatum* Stitz, 1910		HU, IT, TO	Santschi (1924a), Bolton (1980), Van De Perre et al. (2018)
Myrmicinae	*Tetramorium*	*Tetramoriumqualarum* Bolton, 1980		TO	Van De Perre et al. (2018)
Myrmicinae	*Tetramorium*	*Tetramoriumrhetidum* Bolton, 1980		IT	Bolton (1980)
Myrmicinae	*Tetramorium*	*Tetramoriumrotundatum* (Santschi, 1924)		-	Santschi (1924a), Bolton (1980)
Myrmicinae	*Tetramorium*	*Tetramoriumschoutedeni* Santschi, 1924	Endemic	MN	Santschi (1924a), Bolton (1980), Hita Garcia et al. (2010)
Myrmicinae	*Tetramorium*	*Tetramoriumsericeiventre* Emery, 1877		BC, HK, HL, HU, IT, KC, KE, KG, KS, SA, SU, TA, TO	Forel (1913a), Forel (1916), Wheeler (1922a), Wheeler (1922b), Santschi (1924a), Santschi (1928b), Bolton (1980), Hita Garcia and Fisher (2011), Hita Garcia and Fisher (2012)
Myrmicinae	*Tetramorium*	*Tetramoriumsetigerum* Mayr, 1901		BU	Wheeler (1922a), Wheeler (1922b), Bolton (1980)
Myrmicinae	*Tetramorium*	*Tetramoriumsetuliferum* Emery, 1895		-	Mbanyana (2013), Mbanyana et al. (2018)
Myrmicinae	*Tetramorium*	*Tetramoriumsimillimum* (Smith, 1851)		KN, KS, TO	Forel (1916), Wheeler (1922a), Wheeler (1922b), Bolton (1980)
Myrmicinae	*Tetramorium*	*Tetramoriumsusannae* Hita Garcia, Fischer & Peters, 2010		IT, KN	Hita Garcia et al. (2010)
Myrmicinae	*Tetramorium*	*Tetramoriumtabarum* Bolton, 1980		IT	Bolton (1980), Hita Garcia and Fisher (2013)
Myrmicinae	*Tetramorium*	*Tetramoriumtalpa* (Bolton, 1976)	Endemic	KL	Bolton (1976)
Myrmicinae	*Tetramorium*	*Tetramoriumtermitobium* Emery, 1908		IT, SA	Emery (1908), Wheeler (1922b), Levieux (1972), Bolton (1980)
Myrmicinae	*Tetramorium*	*Tetramoriumtrimeni* (Emery, 1895)		EQ, TO	Santschi (1935a)
Myrmicinae	*Tetramorium*	*Tetramoriumubangense* Santschi, 1937	Endemic	NU	Santschi (1937a), Bolton (1980)
Myrmicinae	*Tetramorium*	*Tetramoriumuelense* Santschi, 1923		HU	Santschi (1923), Bolton (1976), Hita Garcia and Fisher (2014)
Myrmicinae	*Tetramorium*	*Tetramoriumunicum* Bolton, 1980		NK	Bolton (1980)
Myrmicinae	*Tetramorium*	*Tetramoriumvenator* Hita Garcia & Fisher, 2014		HK, IT, KL	Hita Garcia and Fisher (2014)
Myrmicinae	*Tetramorium*	*Tetramoriumweitzeckeri* Emery, 1895		HK, HU, IT, NK	Forel (1913a), Wheeler (1922b), Weber (1943), Prins (1963), Bolton (1980)
Myrmicinae	*Trichomyrmex*	*Trichomyrmexdestructor* (Jerdon, 1851)	Exotic	HK	Weber (1964), Wetterer (2008a)
Myrmicinae	*Trichomyrmex*	*Trichomyrmexepinotalis* (Santschi, 1923)	Endemic	KC	Santschi (1923), Bolton (1987)
Myrmicinae	*Trichomyrmex*	*Trichomyrmexoscaris* (Forel, 1894)		HL, HU	Forel (1913a), Wheeler (1922b), Finzi (1939), Bolton (1987)
Myrmicinae	*Trichomyrmex*	*Trichomyrmexrobustior* (Forel, 1892)		HU	Wheeler (1922a), Wheeler (1922b)
Ponerinae	*Anochetus*	*Anochetusafricanus* (Mayr, 1865)		HU, KS, LU	Wheeler (1922a), Wheeler (1922b), Santschi (1923), Santschi (1935b), Brown (1978)
Ponerinae	*Anochetus*	*Anochetusbequaerti* Forel, 1913		BU, HK, HL, HU, NK	Forel (1913a), Forel (1916), Wheeler (1922a), Wheeler (1922b), Brown (1978)
Ponerinae	*Anochetus*	*Anochetusfuliginosus* Arnold, 1948		IT	Brown (1978)
Ponerinae	*Anochetus*	*Anochetusmaynei* Forel, 1913		KN	Forel (1913c), Wheeler (1922b), Brown (1978)
Ponerinae	*Anochetus*	*Anochetusobscuratus* Santschi, 1911		KC, KL, KS	Santschi (1923), Brown (1978)
Ponerinae	*Anochetus*	*Anochetuspellucidus* Emery, 1902		IT, MO	Santschi (1923), Brown (1978)
Ponerinae	*Anochetus*	*Anochetuspunctaticeps* Mayr, 1901		HU	Wheeler (1922a), Wheeler (1922b)
Ponerinae	*Anochetus*	*Anochetustraegaordhi* Mayr, 1904		MN, TO	Forel (1916), Wheeler (1922b), Santschi (1923), Brown (1964c), Brown (1978)
Ponerinae	*Bothroponera*	*Bothroponeraancilla* (Emery, 1899)		TO	Wheeler (1922a), Wheeler (1922b)
Ponerinae	*Bothroponera*	*Bothroponerapachyderma* (Emery, 1901)		BC, BU, HU, IT, KL, TO	Santschi (1920a), Wheeler (1922a), Wheeler (1922b), Weber (1943), Joma and Mackay (2020)
Ponerinae	*Bothroponera*	*Bothroponerarubescens* Santschi, 1937		NU	Schmidt and Shattuck (2014), Joma and Mackay (2020)
Ponerinae	*Bothroponera*	*Bothroponerasanguinea* (Santschi, 1920)		-	Joma and Mackay (2020)
Ponerinae	*Bothroponera*	*Bothroponerasoror* (Emery, 1899)		BC, BU, HK, HL, HU, IT, KL, KS, MN, NK, TO	Forel (1913a), Wheeler (1922a), Wheeler (1922b), Santschi (1930a), Santschi (1937a), Weber (1943), Prins (1963), Prins (1964), Joma and Mackay (2017), Van De Perre et al. (2018)
Ponerinae	*Bothroponera*	*Bothroponeratalpa* André, 1890		BU, HU, NK, TO	Wheeler (1922a), Wheeler (1922b), Yamagiwa et al. (1991), Joma and Mackay (2020)
Ponerinae	*Brachyponera*	*Brachyponerasennaarensis* (Mayr, 1862)		BC, BU, HK, HL, HU, KN, NK, SU, TO	Forel (1913a), Forel (1913c), Wheeler (1922a), Wheeler (1922b), Santschi (1923), Prins (1964), Rafinejad et al. (2009), Wetterer (2013a)
Ponerinae	*Centromyrmex*	*Centromyrmexangolensis* Santschi, 1937		IT, LU, TO	Bolton and Fisher (2008a)
Ponerinae	*Centromyrmex*	*Centromyrmexbequaerti* (Forel, 1913)		HK, KL, MA, TO	Forel (1913a), Forel (1913c), Wheeler (1922b), Brown (1963), Bolton and Fisher (2008a)
Ponerinae	*Centromyrmex*	*Centromyrmexdecessor* Bolton & Fisher, 2008		TA, TO	Bolton and Fisher (2008a)
Ponerinae	*Centromyrmex*	*Centromyrmexereptor* Bolton & Fisher, 2008		TO	Bolton and Fisher (2008a)
Ponerinae	*Centromyrmex*	*Centromyrmexfugator* Bolton & Fisher, 2008		KN	Bolton and Fisher (2008a)
Ponerinae	*Centromyrmex*	*Centromyrmexpraedator* Bolton & Fisher, 2008	Endemic	KL	Bolton and Fisher (2008a)
Ponerinae	*Centromyrmex*	*Centromyrmexsellaris* Mayr, 1896		HK, HU, IT, SK	Weber (1949b), Weber (1964), Bolton and Fisher (2008a)
Ponerinae	*Euponera*	*Euponerabrunoi* (Forel, 1913)		HK	Santschi (1933), Brown (1963)
Ponerinae	*Euponera*	*Euponerasjostedti* (Mayr, 1896)		BC	Wheeler (1922a), Wheeler (1922b)
Ponerinae	*Hypoponera*	*Hypoponeracamerunensis* (Santschi, 1914)		IT	Bolton and Fisher (2011)
Ponerinae	*Hypoponera*	*Hypoponeracoeca* (Santschi, 1914)		TO	Forel (1916), Wheeler (1922b)
Ponerinae	*Hypoponera*	*Hypoponeradulcis* (Forel, 1907)		IT	Bolton and Fisher (2011)
Ponerinae	*Hypoponera*	*Hypoponerainaudax* (Santschi, 1919)		IT, TO	Santschi (1919c), Wheeler (1922b), Braet and Taylor (2008), Bolton and Fisher (2011)
Ponerinae	*Hypoponera*	*Hypoponeramolesta* Bolton & Fisher, 2011		IT	Bolton and Fisher (2011)
Ponerinae	*Hypoponera*	*Hypoponerapunctatissima* (Roger, 1859)		BC, HK, HU, SK, TO	Forel (1916), Wheeler (1922b), Santschi (1933), Delabie and Blard (2002), Bolton and Fisher (2011)
Ponerinae	*Hypoponera*	*Hypoponerasegnis* Bolton & Fisher, 2011		SK	Bolton and Fisher (2011)
Ponerinae	*Hypoponera*	*Hypoponeraursa* (Santschi, 1924)		NK, SK	Santschi (1924a), Santschi (1933), Santschi (1935b), Bolton and Fisher (2011)
Ponerinae	*Leptogenys*	*Leptogenysankhesa* Bolton, 1975	Endemic	BC	Bolton (1975b)
Ponerinae	*Leptogenys*	*Leptogenyscamerunensis* Stitz, 1910		BU, MN, TO	Forel (1916), Wheeler (1922a), Wheeler (1922b), Bolton (1975b)
Ponerinae	*Leptogenys*	*Leptogenysconradti* Forel, 1913		HK	Santschi (1937a)
Ponerinae	*Leptogenys*	*Leptogenyscrustosa* Santschi, 1914		HK, IT	Santschi (1937a), Bolton (1975b)
Ponerinae	*Leptogenys*	*Leptogenysergatogyna* Wheeler, 1922		HU	Wheeler (1922a), Wheeler (1922b), Bolton (1975b)
Ponerinae	*Leptogenys*	*Leptogenysexcellens* Bolton, 1975	Endemic	TO	Bolton (1975b)
Ponerinae	*Leptogenys*	*Leptogenysferrarii* Forel, 1913		HK, TA	Santschi (1915), Wheeler (1922b), Bernard (1953), Bolton (1975b)
Ponerinae	*Leptogenys*	*Leptogenysintermedia* Emery, 1902		KL, SK	Stitz (1911), Wheeler (1922b), Santschi (1926a), Bolton (1975b)
Ponerinae	*Leptogenys*	*Leptogenysmaxillosa* (Smith, 1858)		HK	Bolton (1975b)
Ponerinae	*Leptogenys*	*Leptogenysravida* Bolton, 1975	Endemic	SK	Bolton (1975b)
Ponerinae	*Leptogenys*	*Leptogenysstrator* Bolton, 1975	Endemic	NK	Bolton (1975b)
Ponerinae	*Leptogenys*	*Leptogenystrilobata* Santschi, 1924		BC	Santschi (1924a), Bolton (1975b)
Ponerinae	*Loboponera*	*Loboponeranasica* (Santschi, 1920)		-	Levieux (1972)
Ponerinae	*Loboponera*	*Loboponeratrica* Bolton & Brown, 2002		IT	Fisher (2006)
Ponerinae	*Loboponera*	*Loboponeravigilans* Bolton & Brown, 2002		KL	Bolton and Brown (2002)
Ponerinae	*Megaponera*	*Megaponeraanalis* (Latreille, 1802)		BC, BU, HK, HL, HU, IT, KS, MA, MO, NK, NU, SA, SK, SU, TO	Forel (1911b), Stitz (1911), Forel (1913a), Forel (1913c), Stitz (1916), Wheeler (1922a), Wheeler (1922b), Santschi (1923), Santschi (1930a), Prins (1963), Weber (1964), Van De Perre et al. (2018)
Ponerinae	*Megaponera*	*Megaponeraanalisrapax* Santschi, 1914		BC	Santschi (1923)
Ponerinae	*Megaponera*	*Megaponeraanalistermitivora* (Santschi, 1930)		BC	Santschi (1930a)
Ponerinae	*Mesoponera*	*Mesoponeracaffraria* (Smith, 1858)		-	Prins (1964)
Ponerinae	*Mesoponera*	*Mesoponeraingesta* (Wheeler, 1922)		BU, HU, MA	Wheeler (1922a), Wheeler (1922b), Santschi (1930a), Weber (1943)
Ponerinae	*Mesoponera*	*Mesoponerascolopax* (Emery, 1899)		SU	Stitz (1916), Wheeler (1922b)
Ponerinae	*Mesoponera*	*Mesoponerasubiridescens* (Wheeler, 1922)		BU, HU, NK	Wheeler (1922a), Wheeler (1922b), Weber (1943), Yamagiwa et al. (1991)
Ponerinae	*Odontomachus*	*Odontomachusassiniensis* Emery, 1892		BC, BU, HU, KN, MA, NK, SU, TO	Forel (1910a), Stitz (1910), Emery (1911), Forel (1913a), Stitz (1916), Wheeler (1922a), Wheeler (1922b), Weber (1943), Brown (1976), Yamagiwa et al. (1991)
Ponerinae	*Odontomachus*	*Odontomachustroglodytes* Santschi, 1914		IT, NK, TO	Wheeler (1922a), Wheeler (1922b), Yamagiwa et al. (1991), Fisher and Smith (2008)
Ponerinae	*Paltothyreus*	*Paltothyreustarsatus* (Fabricius, 1798)		BC, BU, EQ, HK, HU, IT, KN, KS, MA, MN, NK, SA, SU, TO	Forel (1909), Stitz (1911), Forel (1913a), Forel (1913c), Stitz (1916), Wheeler (1922a), Wheeler (1922b), Yamagiwa et al. (1991)
Ponerinae	*Paltothyreus*	*Paltothyreustarsatusmedianus* Santschi, 1919		SU	Santschi (1919c), Wheeler (1922b)
Ponerinae	*Parvaponera*	*Parvaponeradarwiniiafricana* (Forel, 1909)		BC, TO	Forel (1909), Wheeler (1922a), Wheeler (1922b), Santschi (1935b)
Ponerinae	*Phrynoponera*	*Phrynoponerabequaerti* Wheeler, 1922		HU, IT	Wheeler (1922a), Wheeler (1922b), Maes and Mackay (1993), Bolton and Fisher (2008a), Bolton and Fisher (2008b),Braet and Taylor (2008)
Ponerinae	*Phrynoponera*	*Phrynoponeragabonensis* (André, 1892)		BU, HU, IT, KL, KN, NK, TA, TO	Santschi (1919c), Wheeler (1922a), Wheeler (1922b), Menozzi (1942), Weber (1943), Brown (1950a), Bolton and Fisher (2008a), Bolton and Fisher (2008b)
Ponerinae	*Phrynoponera*	*Phrynoponerasveni* (Forel, 1916)		HU, TA	Forel (1916), Wheeler (1922a), Wheeler (1922b), Bolton and Fisher (2008a), Bolton and Fisher (2008b)
Ponerinae	*Platythyrea*	*Platythyreaarnoldi* Forel, 1913		MA	Santschi (1937a)
Ponerinae	*Platythyrea*	*Platythyreaconradti* Emery, 1899		KC, MN, TO	Forel (1915), Forel (1916), Wheeler (1922a), Wheeler (1922b), Santschi (1930a), Santschi (1933), Brown (1975), Van De Perre et al. (2018)
Ponerinae	*Platythyrea*	*Platythyreacribrinodis* (Gerstäcker, 1859)		HK	Weber (1943)
Ponerinae	*Platythyrea*	*Platythyreafrontalis* Emery, 1899		KS	Santschi (1933)
Ponerinae	*Platythyrea*	*Platythyreagracillima* Wheeler, 1922		HU, TO	Wheeler (1922a), Wheeler (1922b), Santschi (1937a)
Ponerinae	*Platythyrea*	*Platythyrealamellosa* (Roger, 1860)		MA	Forel (1913c), Wheeler (1922b), Prins (1963)
Ponerinae	*Platythyrea*	*Platythyreamodesta* Emery, 1899		BC, MA, SU, TO	Forel (1916), Stitz (1916), Wheeler (1922b), Brown (1975), Van De Perre et al. (2018)
Ponerinae	*Platythyrea*	*Platythyreaschultzei* Forel, 1910		HK, HL, MA	Forel (1913a), Wheeler (1922b), Brown (1975)Brown 1975
Ponerinae	*Plectroctena*	*Plectroctenacristata* Emery, 1899		BU, HU, KS, MO	Wheeler (1922a), Wheeler (1922b), Santschi (1924b), Bolton (1974b), Bolton and Brown (2002)
Ponerinae	*Plectroctena*	*Plectroctenadentata* Santschi, 1912		-	Bolton (1974b)
Ponerinae	*Plectroctena*	*Plectroctenalaevior* Stitz, 1924	Endemic	SK	Santschi (1924b), Bolton (1974b), Bolton and Brown (2002)
Ponerinae	*Plectroctena*	*Plectroctenalatinodis* Santschi, 1924		KN	Santschi (1924b), Bolton (1974b), Bolton and Brown (2002)Bolton and Brown 2002
Ponerinae	*Plectroctena*	*Plectroctenamandibularis* Smith, 1858		EQ, HK, HL, LU, NK, TA	Forel (1909), Forel (1913a), Wheeler (1922b), Santschi (1924b), Bolton (1974b)
Ponerinae	*Plectroctena*	*Plectroctenaminor* Emery, 1892		BC, BU, EQ, HK, KL, KN, TO	Stitz (1910), Forel (1916), Wheeler (1922a), Wheeler (1922b), Santschi (1924b), Bolton (1974b), Bolton and Brown (2002)
Ponerinae	*Plectroctena*	*Plectroctenaugandensis* Menozzi, 1932		EQ	Bolton (1974b)
Ponerinae	*Psalidomyrmex*	*Psalidomyrmexprocerus* Emery, 1901		BU, HU, IT, KN, NK	Wheeler (1922a), Wheeler (1922b), Santschi (1937a), Bolton (1975a), Bolton and Brown (2002)
Ponerinae	*Psalidomyrmex*	*Psalidomyrmexreichenspergeri* Santschi, 1913		BU	Wheeler (1922a), Wheeler (1922b), Bolton (1975a)
Ponerinae	*Psalidomyrmex*	*Psalidomyrmexwheeleri* Santschi, 1923		BU, HU, IT, NU	Bolton (1975a), Bolton and Brown (2002)
Proceratiinae	*Discothyrea*	*Discothyreadamato* Hita-Garcia & Lieberman, 2019		NK	Hita Garcia et al. (2019)
Proceratiinae	*Discothyrea*	*Discothyreamixta* Brown, 1958		NK	Hita Garcia et al. (2019)
Proceratiinae	*Discothyrea*	*Discothyreaoculata* Emery, 1901		BC, IT, KN	Hita Garcia et al. (2019)
Proceratiinae	*Discothyrea*	*Discothyreawakanda* Hita-Garcia & Lieberman, 2019	Endemic	NK	Hita Garcia et al. (2019)
Pseudomyrmecinae	*Tetraponera*	*Tetraponeraaethiops* Smith, 1877		EQ, HU, IT, KS, MA, NK, SA, SK, SU, TO	Stitz (1911), Forel (1913a), Forel (1913c), Forel (1916), Stitz (1916), Wheeler and Bailey (1920), Wheeler (1922a), Wheeler (1922b), Van De Perre et al. (2018), Ward (2022)
Pseudomyrmecinae	*Tetraponera*	*Tetraponeraanthracina* (Santschi, 1910)		BC, EQ, IT, KE, KG, KL, KN, KS, MA, MO, NK, TA, TO	Forel (1911b), Forel (1913c) , Forel (1916), Wheeler (1922a), Wheeler (1922b), Santschi (1928a), Santschi (1933), Terron (1968), Van De Perre et al. (2018), Ward (2022)
Pseudomyrmecinae	*Tetraponera*	*Tetraponeracortina* Ward, 2022		TO	Ward (2022)
Pseudomyrmecinae	*Tetraponera*	*Tetraponeralatifrons* (Emery, 1912)		BC, HU, KS, SA	Forel (1913c), Wheeler and Bailey (1920), Wheeler (1922a), Wheeler (1922b), Ward (2022)
Pseudomyrmecinae	*Tetraponera*	*Tetraponeramocquerysi* (André, 1890)		BC, HK, HU, IT, KN, KS, LU, MA, MO, NK, SK, TA, TO	Forel (1911b), Forel (1913c), Forel (1916), Santschi (1920a), Wheeler (1922a), Wheeler (1922b), Santschi (1928a), Santschi (1935b), Van De Perre et al. (2018), Ward (2022)
Pseudomyrmecinae	*Tetraponera*	*Tetraponeranatalensis* (Smith, 1858)		HK, KS, SK, TA	Stitz (1911), Wheeler (1922b), Ward (2022)
Pseudomyrmecinae	*Tetraponera*	*Tetraponeraophthalmica* (Emery, 1912)		BC, KS, MO, NK, SK, TO	Emery (1912), Forel (1916), Wheeler (1922a), Wheeler (1922b), Santschi (1928a), Baroni Urbani (1977), Ward (2006)
Pseudomyrmecinae	*Tetraponera*	*Tetraponerapumila* Ward, 2022		HK, NK	Ward (2022)
Pseudomyrmecinae	*Tetraponera*	*Tetraponeratessmanni* (Stitz, 1910)		HU, IT, TO	Santschi (1919c), Wheeler and Bailey (1920), Wheeler (1922a), Wheeler (1922b), Brown (1950a), Baroni Urbani (1977), Ward (2022)
