# Supplementary material for: Overview of myrmecological studies and a checklist of the ants (Hymenoptera, Formicidae) of the Democratic Republic of Congo
Source: Biodivers Data J. 2024 Oct 18;12:e132915. doi: 10.3897/BDJ.12.e132915 (PMC11512105; doi:10.3897/BDJ.12.e132915)
Supplement: Supplementary material 1 — List of protected areas in DRC [file bdj-12-e132915-s001.docx]

**Additional information**

Table 4. List of protected areas in DRC. Number in parenthesis represents the approximate area in km^2^. “*” denotes UNESCO wetland of international importance.

| **National Park** | **UNESCO Biosphere Reserve** | **Nature Reserve** | **Hunting Reserve** | **Wildlife Reserve** | **Scientific**  **Reserve** | **Community Reserve** |
| --- | --- | --- | --- | --- | --- | --- |
| Garamba (5,200) | Lufira  (687)* | Abumonbazi  (5,726) | Bili-Uere  (32,748) | Bomu  (4.125) | Luo  (225) | Lyondji Bonobo  (1,030) |
| Kahuzi-Biega  (6,689) | Luki  (362) | Itombwe  (6,009) | Bombo Lumene  (2,168) | Okapi  (14.000) |  |  |
| Kundelungu  (7.600) | Yangambi  (2,350) | Kisimba Ikobo Primate  (963) | Bomu  (4,126) |  |  |  |
| Lomami  (8,879) |  | Lake Tshangalele  (365) | Bushimaie  (4,369) |  |  |  |
| Maiko  (10,886) |  | Lomako-Yokokala  (3,602) | Gangala-na-Bodio  (9,829) |  |  |  |
| Mangroves Marine  (668)* |  | Mangai  (1,903) | Luama-Katanga  (2,308) |  |  |  |
| Salonga  (36.000) |  | Mangrove  (1,000) | Luama-Kivu  (3,900) |  |  |  |
| Upemba  (13,000) |  | Ngiri Triangle  (1.000)* | Lubudi-Sampwe  (3,489) |  |  |  |
| Virunga  (8.090)* |  | N'Sele  (200) | Maika-Penge  (1,499) |  |  |  |
|  |  | Sankuru Nature Reserve  (30.570) | Rubi-Tele  (6,191) |  |  |  |
|  |  | Tayna  (893) | Rutshuru  (661) |  |  |  |
|  |  | Tumba-Lediima  (7,412) | Swa-Kibula  (1,004) |  |  |  |
